# Variation in floral form of CRISPR knock-outs of the poplar homologs of *LEAFY* and *AGAMOUS* after FT heat-induced early flowering

**DOI:** 10.1093/hr/uhad132

**Published:** 2023-06-29

**Authors:** Amy L Klocko, Estefania Elorriaga, Cathleen Ma, Steven H Strauss

**Affiliations:** Department of Biology, University of Colorado Colorado Springs, Colorado Springs, CO 80918, USA; Department of Forest Ecosystems and Society, Oregon State University, Corvallis, OR 97331, USA; Department of Forest Ecosystems and Society, Oregon State University, Corvallis, OR 97331, USA; Department of Forest Ecosystems and Society, Oregon State University, Corvallis, OR 97331, USA

## Abstract

Plant migration and gene flow from genetically modified or exotic trees to nearby lands or by crossing with wild relatives is a major public and regulatory concern. Many genetic strategies exist to mitigate potential gene flow; however, the long delay in onset of flowering is a severe constraint to research progress. We used heat-induced FT overexpression to speed assessment of the expected floral phenotypes after CRISPR knockout of poplar homologs of the key floral genes, *LEAFY* and *AGAMOUS*. We selected events with previously characterized CRISPR-Cas9 induced biallelic changes then re-transformed them with the *Arabidopsis thaliana FLOWERING LOCUS T (AtFT*) gene under control of either a strong constitutive promoter or a heat-inducible promoter. We successfully obtained flowering in both a male and female clone of poplar, observing a wide range of inflorescence and floral forms among flowers, ramets, and insertion events. Overall, flowers obtained from the selected *LFY* and *AG* targeted events were consistent with what would be predicted for loss-of-function of these genes. *LFY*-targeted events showed small catkins with leaf-like organs, *AG*-targeted events had nested floral organs consistent with reduction in floral determinacy and absence of well-formed carpels or anthers. These findings demonstrate the great developmental plasticity of *Populus* flowers during genetically accelerated flowering, which may be of horticultural value. They also provide an informative early view of floral phenotypes and apparent sterility from knockouts of both these gene targets.

## Introduction

The movement or gene flow from genetically modified, domesticated, or potentially invasive plant species to wild lands or into sexually compatible relatives is a substantial public and regulatory concern. Many forestry and horticultural species are grown outside of their native range, and some have become invasive due to their ability to propagate within those environments [1]. Many strategies have been developed to mitigate potential sexual dispersal and spread, both by conventional and transgenic or gene-editing approaches (reviewed in [2]. These include natural cleistogamy, identifying naturally sterile varieties, creation of sterile hybrids, using sterile polyploids, and engineered sterility via transgenic or gene-editing methods. The mechanisms of engineered sterility include pollen or organ ablation, gene suppression by RNA interference (RNAi), or targeted gene editing of essential flowering genes, among others.

As knowledge of the molecular biology of flowering has grown, methods that seek to suppress or eliminate the functions of key, highly conserved floral genes for genetic containment have grown. For example, RNAi directed against the floral genes *LEAFY* (*LFY*) and *AGAMOUS* (*AG*) have been at least partly successful in attenuating fertility in apple [3], sweetgum [4], and poplar [5–7]. Where the goal is complete disruption of gene function, CRISPR knockouts have become a powerful tool for genetic containment. CRISPR is known to work with high efficiency in almost any animal or plant species, and there are many possible applications in woody species [8, 9]. Our previous work has established that CRISPR-Cas9 gene editing of floral development genes is efficient at creating bi-allelic mutations in target genes in both Eucalyptus [10, 11] and poplar [12]. In the former case, we also demonstrated the use of FT-induced rapid flowering to speed the evaluation of floral form in Eucalyptus, for which constitutive overexpression worked well. However, as discussed below this method has low efficiency in poplar, prompting us to employ a heat induction system to stimulate early flowering.

Many strategies have been tried to obtain faster flowering in tree species. These include, altering the photoperiod [13], hormone and fertilizer treatments [14], finding and breeding rare naturally early-flowering individuals [15], RNAi-based suppression of key floral genes [16, 17], and increased expression of key floral regulatory genes by either viral expression or stable transformation. Viral induced expression has been used successfully to induce early-flowering in domestic apple, and resulted in formation of viable pollen, a shortened breeding period, and the successful identification of virus-free progeny [18].

Most research on genetically-accelerated flowering relies on stable transformation, for which a variety of promoter and gene combinations have been employed to overexpress floral inductive genes (Table 1). Nearly all published reports include successful early flowering, which indicates the robustness of this approach, and likely the tendency to publish positive results. For work within *Populus*, different clones showed different degrees of floral induction, even for the same genetic construct tested at the same location [33, 40], (Supplemental Table S1). Sometimes, in addition to the desired early flowering, there were unintended impacts on vegetative form. For example, the use of the strong viral-derived 35S promoter to control target gene expression often resulted in shortened plant stature, as was observed for poplar, plum, and eucalypts [10, 21, 28, 32, 41]. Therefore, it can be challenging to parse out any vegetative differences if this accelerated flowering system is used to assess vegetative impacts of genetic containment systems. Our prior study of CRISPR-Cas targeting of *LFY* in Eucalyptus used two separate populations for this very reason; one set of trees with CRISPR-derived gene edits and *Arabidopsis thaliana FLOWERING LOCUS T (AtFT)* overexpression was used for evaluation of floral form, while CRISPR edited trees (in the absence of genetically accelerated flowering) was used for the analysis of overall juvenile tree form and growth [10].

**Table 1:** Constructs used to induce early flowering in trees by stable transformation.

**Promoter**	**Gene**	**Gene source**	**Tree transformed**	**Floral outcome**	**Reference**
*constitutive*					
35S	*BpMADS4*	*Betula pendula* Roth	*Malus domestica*	Flowering	[19]
35S	*AtFT*	*Arabidopsis thaliana*	*M. domestica*	No flowering	[20]
35s	*AtFT*	*A. thaliana*	*Eucalyptus grandis x E. urophylla* clone SP7	Flowering	[21]
35S	*AtFT*	*A. thaliana*	*Populus tremula L. x Populus* *tremuloides Michx., clone T89 P. tremula L., clone W52*	Flowering^*^	[22]
35S	*AtAP1*	*A. thaliana*	*Fortunella crassifolia* Swingle	Flowering	[23]
35S	*AtAP1*	*A. thaliana*	*Citrus sinensis L. Osbeck × Poncirus trifoliata* L. Raf.	Flowering	[24]
35S	*CiFT*	*Citrus unshiu*	*P. trifoliata* L. Raf	Flowering	[25]
35S	*CiFT*	*C. unshiu*	*Pyrus communis L.* “Ballade”	Flowering	[26]
35S	*PtFT1*	*Populus trichocarpa*	*P. tremula x P. tremuloides*	Flowering	[27]
35S	*PtFT1*	*P. trichocarpa*	*Prunus domestica*	Flowering	[28]
35S	*MtFTa1*	*Medicago truncatula*	*Olea europaea L.*	Flowering	[29]
35S	*MdFT1*	*M. domestica*	*P. tremula* clone W52	Flowering	[30]
35A	*AtLFY*	*A. thaliana*	*P. tremula x P. tremuloides*	Flowering	[31]
35S	*AtLFY*	*A. thaliana*	*P. tremula L. x P. tremuloides Michx., clone T89 P. tremula L., clone W52*	Flowering	[22]
35S	*AtLFY*	*A. thaliana*	*P. tremula x P. tremuloides, P. tremula*	Flowering	[32]
35S	*AtLFY*	*A. thaliana*	*C. sinensis L. Osbeck × P. trifoliata* L. Raf.	Flowering	[24]
35S	*AtLFY*	*A. thaliana*	*P. tremula x Populus* * alba clone 717 P. tremula x P. tremuloides clone 353*	Flowering	[33]
35S	*PtLFY*	*P. trichocarpa*	*P. tremula x Populus alba clone 717 P. tremula x P. tremuloides clone 353*	Flowering^**^	[33]
35S	*rolC*	*Agrobacterium rhizogenes*	*P. tremula P. tremuloides*, clone Esch5	Flowering	[34]
35S	*rolC*	*A. rhizogenes*	*P. tremula*, clone W52	No flowering	[34]
35S	*rolC*	*A. rhizogenes*	*P. tremula*, clone Brauna11	No flowering	[34]
35S	*CcFT1*	*Citrus clementina*	*C. sinensis* Osb. × *P. trifoliata* L. Raf.	No flowering	[35]
35S	*CcFT3*	*Citrus C. clementina*	*C. sinensis* Osb. × *P. trifoliata* L. Raf.	Flowering^+^	[35]
35S	*ToFT*	*Citrus trifoliata*	*C. trifoliata*	Flowering	[36]
35S	*AtFT*	*A. thaliana*	*C. trifoliata*	Flowering	[36]
35S	*PcFT2*	*P. communis L.*	*M. domestica*	No Flowering	[37]
NOS	*CcFT1*	*C. clementina*	*C. sinensis* Osb. × *P. trifoliata* L. Raf.	No flowering	[35]
NOS	*CcFT3*	*Citrus C. clementina*	*C. sinensis* Osb. × *P. trifoliata* L. Raf.	Flowering^++^	[35]
409S	*AtFT*	*A. thaliana*	*E. grandis x E. urophylla* clone SP7	Flowering	[21]
*Tissue-specific (phloem limited)*					
AtSUC2	*CcFT1*	*C. clementina*	*C. sinensis* Osb. × *P. trifoliata* L. Raf.	No flowering	[35]
AtSUC2	*CcFT3*	*Citrus C. clementina*	*C. sinensis* Osb. × *P. trifoliata* L. Raf.	Flowering	[35]
*Heat inducible*					
HSP	*AtFT*	*A. thaliana*	*P. tremula x P. tremuloides P. tremula*	Flowering	[38]
HSP	*AtFT*	*A. thaliana*	*P. tremula L*., clone W7	Flowering	[39]
HSP	*AtFT*	*A. thaliana*	*P. tremula L. x P. tremuloides Michx., clone T89 P. tremula L., clone W52*	Flowering	[22]
HSP	*AtFT*	*A. thaliana*	*P. tremula x P. tremuloides clone 353 P. tremula x P. alba clone 717*	Flowering	[40]
HSP	*PtFT1*	*P. trichocarpa*	*P. tremula x P. tremuloides clone 353 P. tremula x P. alba clone 717*	Flowering	[40]
HSP	*PtFT1*	*P. trichocarpa*	*E. grandis x E. urophylla* clone SP7	Flowering	[21]
HSP	*PtFT2*	*P. trichocarpa*	*P. tremula x P. tremuloides clone 353 P. tremula x P. alba clone 717*	Flowering^**^	[40]
HSP	*AtLFY*	*A. thaliana*	*P. tremula L. x P. tremuloides Michx., clone T89 P. tremula L., clone W52*	No flowering	[22]
HSP	*CcFT1*	*C. clementina*	*C. sinensis* Osb. × *P. trifoliata* L. Raf.	No flowering	[35]
HSP	*CcFT3*	*C. clementina*	*C. sinensis* Osb. × *P. trifoliata* L. Raf.	No flowering	[35]

Examples of work in *Populus* species are shaded in grey*.*

^+^flowered *in vitro.*

^++^plants flowered once then stopped.

a 1 event flowered

b clone 353 flowered while clone 717 did not flower

Here, our main goal was to use genetically-accelerated flowering to obtain an early view of floral morphology on male and female clones of poplar that have been CRISPR-mutated at the poplar *LFY* or *AG* genes. *LFY* and *AG* are known key floral development genes and are highly conserved across plant species. Our prior work using RNA interference (RNAi) to suppress *LFY* and *AG* in poplar showed modification of female floral form and loss of seed formation under natural flowering in field conditions [10, 21, 42]. We generated CRISPR-Cas9 constructs targeting either the single *LFY* or both *AG*-like genes, and successfully obtained a large number of independent events with bi-allelic targeting of one or both target genes [12]. Selected knockout events were then retransformed with *FT* transgenes. In prior work, we tested a variety of floral induction constructs to inform our choice of promoter and gene [40], for which a heat-inducible *AtFT* system was the most efficient. Such an approach should reduce the strong impacts of *FT* overexpression on vegetative form [19, 21, 28, 29, 35]. This HSP-*AtFT* system has been previously used to evaluate male sterility in poplars [22, 38]. One challenge to heat-induced flowering is that a change to lower temperatures is needed to obtain viable pollen formation [43]. However, lower temperatures can also lead to floral reversion to vegetative structures, which complicates evaluation of floral form [40]. Therefore, we focused our efforts on evaluating overall floral form without attempting to use lower temperatures to achieve viable pollen—especially as both *LFY* and *AG* knockouts are expected to lack any pollen production. We report a high rate of successful flowering induction, and in knockouts we observed the expression of a wide range of floral phenotypes that are congruent with expectation for these genes and suggest that there will be a high rate of floral sterility in normally flowering trees.

## Results

### Optimizing early floral induction

**Figure 1:**
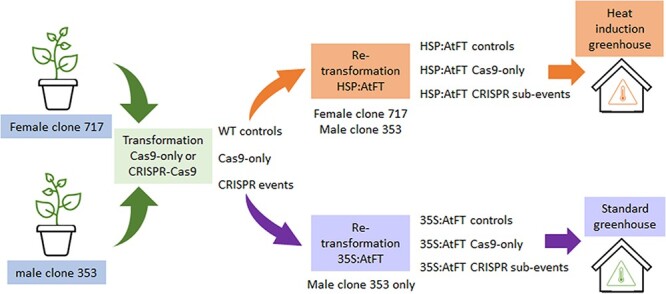
Experimental overview. A schematic representation of the overall workflow. A female and male clone of hybrid poplar were selected as the initial starting materials. Both clones underwent initial transformation with either a Cas9-only control vector (lacking guide RNAs) or a CRISPR-Cas9 vector (including guide RNAs, called Cas9-only control). These events were then characterized by DNA sequencing of the target sites to identify allelic changes. Selected events from both female clone 717 and male clone 353 underwent re-transformation with the vector HSP:AtFT to obtain doubly transformed sub-events for a heat-induced floral study. Selected events from male clone 353 also underwent re-transformation with the vector 35S:AtFT to induce flowering in a standard greenhouse.

We tested four different constructs for inducing early-flowering. We achieved flowering rates ranging from 0% to 48.5% of events (independent gene insertions) within each of the two tested clones (Supplemental Table S2). The promoter and gene combination which gave the highest rate of flowering for both clones 717 and 353 was HSP:*AtFT*. Overall, female clone 717 had a lower rate of flowering than male clone 353. Of the two different *Populus tremula x Populus tremuloides* clones, we achieved flowering with clone 353 but not with clone T89. From these findings, we created an experimental plan to induce early-flowering in selected CRISPR trees in clones 353 and 717 by re-transforming selected events with either constitutive or heat-inducible floral constructs (Figure 1).

### CRISPR-Cas9 event selection for re-transformation with floral-inducing constructs

**Figure 2:**
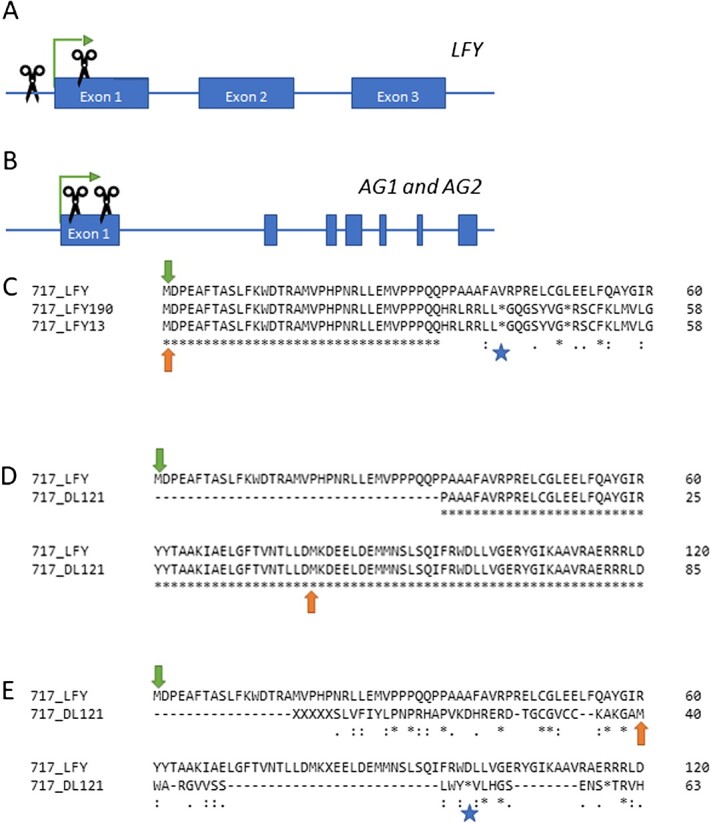
Locations of gene edits in *LFY* and *AG* genes and predicted peptides in LFY. (A) Guide RNA target sites in *LFY* are shown with scissors. Boxes show exons, thin lines show non-coding regions. Green arrows indicate start codons. Note that the first target site in *LFY* occurs prior to exon 1. (B) Guide RNA target sites in both *AG1* and *AG2* are shown with scissors. (C) Predicted LFY peptides in clone 717. Events 190 and 13 have a single guide RNA designed to target Exon 1. Note that while the start of the LFY peptide is conserved, the single deletion mutations cause a frame shift and early stop codon. Green and orange arrows indicate first methionine (M) in WT and edited alleles, respectively. Blue star shows the first predicted stop codon in the edited alleles. (D, E) Possible predicted peptides for the 120 bp deletion mutation in event 121. This event lacks the start codon but generates an in-frame deletion. (D) Use of the second in-frame M for LFY results in a peptide prediction missing the first several amino acids but retaining the c-terminus. (E) Use of the first in-exon M results in a very short peptide that shows little similarity to LFY and terminates early. Green and orange arrows indicate first M in WT and edited alleles, respectively. Blue star shows the first predicted stop codon in the edited alleles. Note that all alignments begin with the first M in LFY and show the N-terminus of the protein. LFY events in clone 353 have identical mutations to the 717 events shown here.

A main goal of this work was to induce early flowering in events with known changes to the floral gene *LFY* or to both *AG* genes. We selected a set of previously characterized Cas9 control and CRISPR events in male clone 353 and female clone 717 [12]. Events were chosen that had bi-allelic, putatively knockout mutations (encoding a non-functional protein) in *LFY* or bi-allelic changes in both *AG1* and *AG2* (Figure 2, Supplemental Tables S3 and S4). All chosen *AG* events were generated using two sgRNAs and were named DAG events (for double sgRNA in *AG*). Chosen *LFY* targeting events either had one sgRNA and were named LIC events, or two sgRNAs and were named DL (for double targeting of *LFY*) events. For chosen *LFY* events, the single targeting site was located within Exon 1, while the second sgRNA target site was located prior to the coding region, such that events with large deletions no longer retained the start codon. For the *AG* events chosen, the sgRNA target sites were located within Exon 1. All selected events were used for two approaches (Figure 1). Male clone 353 was selected for a constitutive floral induction approach using the strong promoter 35S driving expression of *AtFT*, allowing for evaluation in a standard greenhouse. Events in both clones were re-transformed with a heat-inducible floral construct for evaluation in a heat-induction greenhouse.

### Floral induction in male clone 353 with 35S:AtFT

For our constitutive floral induction approach, we transformed selected Cas9 and *LFY* events in male clone 353 with the constitutive 35S:*AtFT* construct. Our past work showed that this clone had a higher rate of genetically accelerated floral induction than female clone 717 ([40], Supplemental Table S2). We successfully obtained many re-transformants for analysis (341 ramets from 30 sub-events and 10 WT controls: Supplemental Figure S1). Of these, no flowering was observed for the WT control trees, 16.8% of 35S:*AtFT* flowering control trees produced flowers, and no flowering was observed for any of the 34 *AtFT* sub-events in the Cas9 event background. Retransformation of two selected *LFY* events (DL 143 and DL 106) gave 52.2% and 83.3% flowering by sub-event (Supplemental Figure S1). Microscopy of selected *AtFT* flowering control and flowering sub-events from DL 143 showed the presence of well-formed anthers on the *AtFT* control catkins, while the DL 143 *AtFT* subevents had catkins with underdeveloped fuzzy floral organs, an absence of well-developed anthers, and the presence of many leaf-like structures in the catkins. Due to the typically lower flowering rate for female clone 717 we did not transform events in this clone with the 35S:*AtFT* construct, but instead chose to focus on heat-inducible flowering.

### Floral induction of male clone 353 and female clone 717 with HSP:*AtFT*

Our next approach was to retransform selected events in male clone 353 and female clone 717 with a heat-inducible floral construct HSP:*AtFT*. Here we obtained 250 ramets in clone 353 representing 130 sub-events (Supplemental Table S5), and 170 ramets in clone 717 representing 97 sub-events (Supplemental Table S6). Due to space constraints, trees were tested one clone at a time. We had two adjacent heat-induction greenhouses, designated A and B, and identical sets of trees were present in each greenhouse for each heat induction. For clone 353, the overall flowering rate was 22.4% of all trees, and this rate was similar between the two greenhouses (Supplemental Table S7). For clone 717, the overall flowering rate was 34.1%, with greenhouse A showing 49.4% of trees flowering while greenhouse B showed just 18.1% of trees flowering (Supplemental Table S8).

### Vegetative performance HSP:*AtFT*

We quantified vegetative performance of trees after heat-induced flowering by measuring total tree height. For clone 353, we measured trees twice during the heat-induction period, once in March and once in April. Trees were scored as flowering or non-flowering to allow for detection of any growth differences associated with floral onset. We found that grouping trees by initial event showed similar growth between groups, and that flowering trees grew equally as tall as non-flowering trees (Supplemental Figure S2). For clone 717, we completed the height measurements once during the heat-induced growth. We also found that groups of these trees showed similar growth, and that flowering trees grew equally as tall as did the non-flowering trees (Supplemental Figure S3).

### Flowering and floral phenotypes of male clone 353 with HSP:AtFT

We tested a total of 250 ramets in male clone 353 representing 121 *AtFT* transformation and retransformation events (1 *AtFT* only, 20 *AtFT* in Cas9, and 100 sub-events from selected CRISPR-Cas9 trees: Supplemental Table S5). No flowering was observed prior to heat-induction conditions. After heat induction, of the 250 total ramets 56 (22.4%) flowered, 190 (76.0%) did not flower, and 4 (1.6%) died (Supplemental Table S7). Whole trees appeared in general good health following heat induction (Supplemental Figure S4).

**Figure 3:**
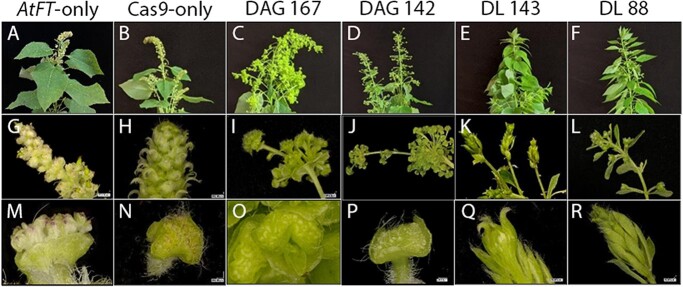
Crown, catkin, and floral morphology of control and knockout genotypes in male clone 353. Tree apexes, catkins, and single flower examples of *AtFT*-only control trees (A, G, and M), Cas9-only *AtFT*-control trees (B, H, N), DAG double biallelic event 167 (C, I, O), DAG double biallelic event 142 (D, J, P), DL biallelic event 143 (E, K, Q), and DL biallelic event 88 (F, L, R).

We used photography followed by microscopy of selected ramets to examine floral morphology (Figure 3). The HSP:*AtFT* control trees typically produced a terminal catkin at the apex of the tree, as well as numerous axial catkins. These catkins had a perianth cup and structures consistent with developing anthers (Figure 3).

**Figure 4:**
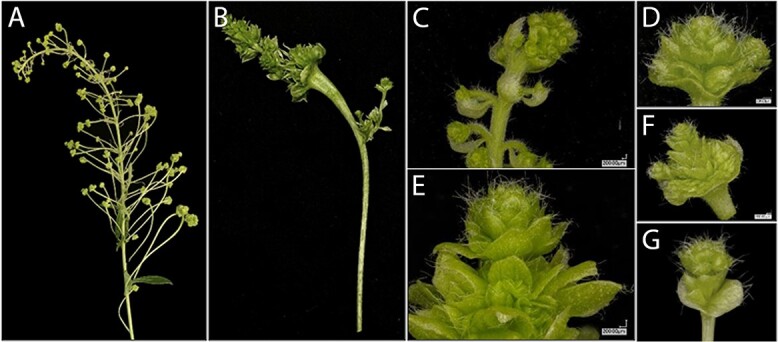
Male *ag* flowers from clone 353 knock-out lines. Inflorescences and close-up view of catkins from DAG double biallelic event 142 (A, C-F) and DAG double biallelic event 167 (B, G). Note the absence of anthers and flat leaf-like appearance of the floral organs.

Cas9-only *AtFT* sub-events were similar in overall appearance to *AtFT* only flowering control events. Occasionally, carpels were observed on catkins of *AtFT* control and Cas9 *AtFT* trees (Supplementary Figure S5). These carpels were typically present in the basal area of the catkin and were surrounded by anthers. Retransformation of *AG* events 142 and 167 led to production of atypical catkins (Figure 4).

**Figure 5:**
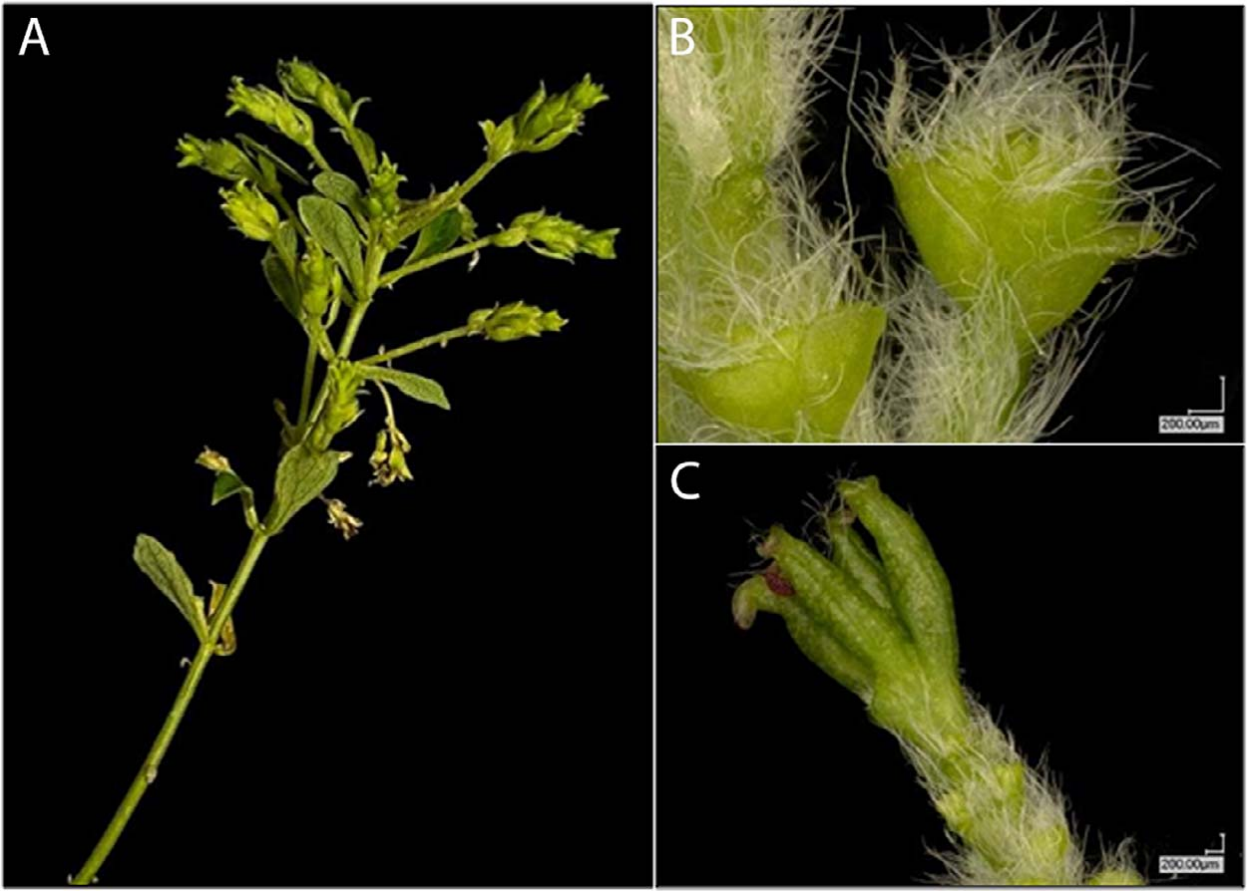
Male *lfy* flowers in a CRISPR knock-out line. Example of inflorescence (A) and catkins (B, C) from DL event 143, tree 221.

In these re-transformation events, catkins were often elongated and contained multiple layers of floral organs. Individual flowers within the inflorescence often had long pedicels. Clusters of small organs in perianth cups were typically green in appearance and these flowers lacked well-developed anthers. Retransformation of *LFY* events 88 and 143 sometimes led to growth of branch-like structures in positions that gave rise to catkins in control events (Figure 5).

Microscopic examination of these structures showed they had many organs that were leaf-like in appearance, with numerous trichomes present and a green coloration (Figure 5). We occasionally observed small regions of reddish colored tissue (Figure 5 panel C) which may be partially developed anthers.

**Figure 6:**
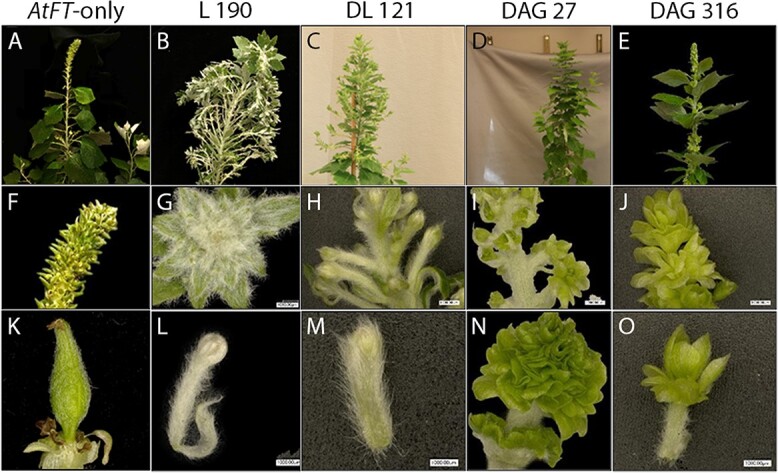
Floral morphology of female clone 717 in control and knockout lines. Tree apexes, catkins, and single flower examples of *AtFT*-only control trees (A, F, K), LFY event 190 (B, G, D), DL event 121 (C, H, M), DAG event 27 (D, I, N), and DAG event 316 (E, J, O).

We tested a total of 170 ramets in female clone 717 representing 97 *AtFT* transformation and re-transformation events (1 *AtFT* only, 13 *AtFT* in Cas9-only, and 83 from selected CRISPR-Cas9 trees, Supplemental Table S6). No flowering was observed prior to heat-induction conditions. After heat induction, of the 170 total ramets 58 (34.1%) flowered, 107 (62.9%) did not flower, and 5 (2.9%) died (Supplemental Table S8). After heat-induction trees from this clone appeared a bit stressed, with yellowing of lower foliage (Supplemental Figure S6). Only three of the nine *AtFT* flowering control trees flowered (Supplemental Table S6). Flowering controls typically had a terminal catkin with numerous carpels (Figure 6).

**Figure 7:**
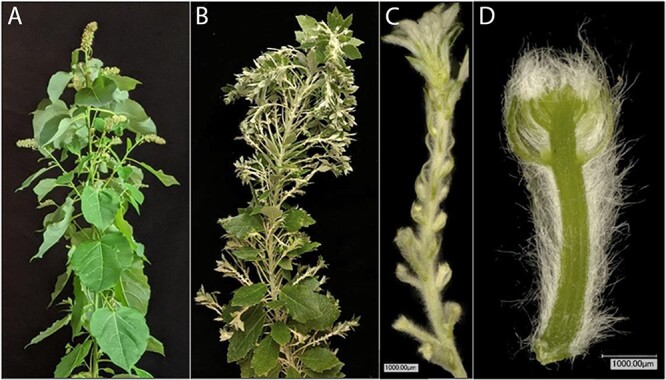
Crown and floral morphology of female *lfy* mutant in clone 717**.** (A) Apexes of flowering control trees typically had a larger terminal catkin and numerous axillary catkins. By contrast, *lfy* knockout mutants such as event 190 tree 39 (B-D) had a very different appearance. (B) The tree apex had numerous projecting branch-like growths in positions typically occupied by catkins. (C) Examination of these structures revealed numerous, fuzzy, underdeveloped potential catkins. (E) Dissection of a single “catkin” showed an absence of carpels or other internal organs.

Re-transformation of *LFY* events 190 and 121 resulted in trees with branch-like structures near the tree apex, which gave these trees a bushy appearance in this location. Microscopic examination of these structures showed small underdeveloped fuzzy organs (Figure 7).

**Figure 8:**
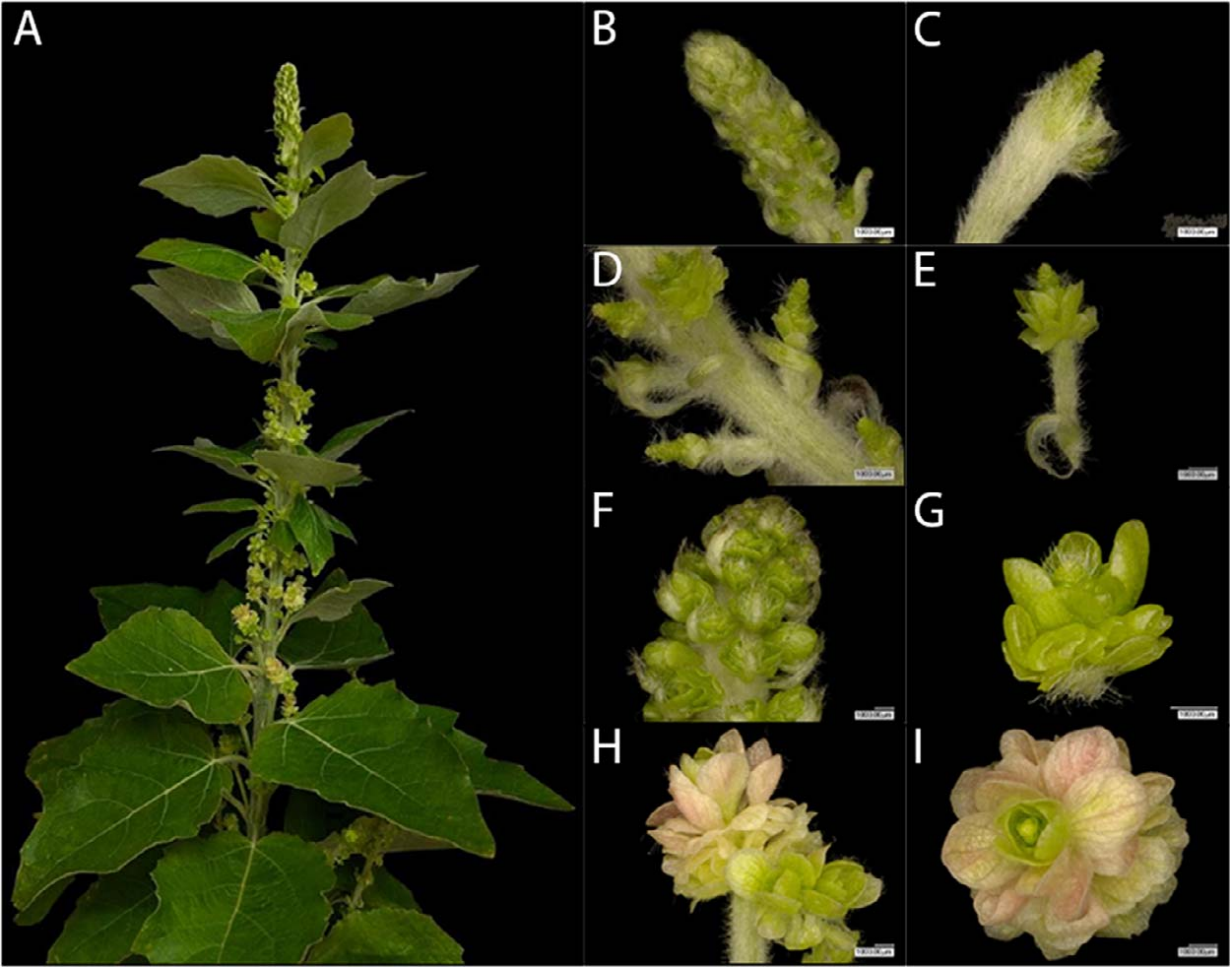
Intra-ramet and intra-catkin variation in floral form in female clone 717. All images are from a single tree of knockout DAG event 316. (A) Highly-variable catkin morphology was observed for the apex of DAG event 316 *AtFT* sub-event 23, ramet 50. (B-I) Microscopy of individual catkins showed phenotype diversity between and within catkins from the same tree. Individual flowers showed loss of well-formed carpels, presence of extra whorls of floral organs, and presence of flattened organs.

While we scored such trees as “flowering,” these organs had no obvious well-developed floral structures. Re-transformation of *AG* events 27 and 316 showed similar outcomes (Figure 8). Here, the trees grew structures that had clusters of flat replicated organs. Microscopic examination of these structures revealed a wide range of phenotypic diversity. The most striking example can be found in the variety of floral forms from a single tree in event 316 (Figure 8). Here, the terminal catkin was somewhat normal in appearance, if underdeveloped. Other catkins and flowers from this same tree showed layers of green, flat floral organs; similar floral structures found in another region of this same trees had a rather ornamental appearance, with layers of pink petal-like organs. This phenotypic variation was also present between trees of the same *AtFT* sub-event, between trees from the same *AG* event but different *AtFT* sub-event, and between trees from different *AG* events (Supplemental Figure S7). All the *AG* knockout trees had replicated floral organs, but the appearance of the organs, in terms of size and color, was variable.

## Discussion

A main goal of this work was to further develop a system for inducing robust early-flowering in both male and female poplar trees. Our initial tests of flowering induction with a variety of constructs showed that male clone 353 typically flowered at a higher rate than female clone 717, which is similar to previous studies of genetically accelerated flowering in greenhouse conditions (Supplemental Table S2, [40]). However, our prior work with a large-scale field trial of natural flowering trees showed that clone 717 flowered at a younger age and higher rate than clone 353 [44]. These findings indicate that clone 353 may be more tractable for genetically accelerated flowering but that this same clone takes longer to reach natural maturity in field tests than clone 717. We selected heat-induced flowering as the primary approach to induce early-flowering in our CRISPR events as this method gave a floral induction rate of 31.0% in clone 717 and 48.5% in clone 353 and avoids pre-heat changes in tree morphology.

We selected CRISPR events that had been previously characterized regarding allele changes in target genes but had yet to undergo floral phenotype evaluation [12]. Chosen events had bi-allelic changes that disrupted predicted peptide formation and were likely to interfere with LFY or AG function (Figure 2, Supplemental Tables S3, S4). Re-transformation of our selected Cas9-only and CRISPR events was successful for both clones, and we obtained a large number of transgenic shoots harboring both the HSP:*AtFT* transgene and the initial Cas9 or CRISPR-Cas9 cassette. As event-to-event differences can be substantial for transgenic plants, we decided to maximize the number of re-transformation events studied rather than choosing numerous ramets per event. The overall floral induction rate of our 353 CRISPR study was 22.4%, which was lower than the 48.5% we found for our initial heat-induction study of this clone. Floral induction of 717 CRISPR events flowered at a rate of 34.1%, which was slightly higher the initial heat-induction test of 31.0%. One possible reason for the lower flowering rate in clone 353 is the timing of this experiment. Clone 353 ramets underwent the large-scale heat induction in February and March, which is a cool and cloudy time of year in western Oregon, USA. The heat-induction greenhouses are located on the building exterior and as such are influenced by the external environment, such as ambient air temperature and sun exposure. By contrast, clone 717 underwent large-scale heat induction starting in August, when outside temperatures are generally very warm (often above 30°C in afternoons) and clouds are sparse.

One advantage to heat-induced flowering is it avoids the striking branchy and dwarf phenotypes seen when strong constitutive promoters driving *FT* are used [21, 28]. With our heat-induced *FT* system, both clones showed no significant difference in tree size between heat-induced flowering and non-flowering trees (Supplemental Figures S2, S3). Observations of overall tree form showed that most CRISPR trees were similar to *AtFT* flowering control trees following heat induction (Supplemental Figures S4, S6). One exception was some ramets with CRISPR targeting *LFY* that developed a bushy appearance at the apex (Figure 7). This extra growth appeared to be due to the presence of branch-like vegetative structures in positions where catkins typically develop.

A primary focus of this work was determining impacts of CRISPR targeting of key floral development genes on floral form and fertility. We did not directly assess floral fertility (e.g. pollen formation and germination), as producing fertile heat-induced poplar flowers requires highly specific growth conditions [43]. We instead focused on floral morphology changes relevant to whether *any* gametogenic floral organs would form at all. We found that, in general, targeting of *LFY* led to small, underdeveloped flowers in both male clone 353 and female clone 717 (Figures 5, 7). These flowers were often highly leaf-like or branch-like, where it could sometimes be a challenge to determine the type of organ being formed. These *LFY* targeted trees showed an absence of well-formed anthers or carpels, no evidence of male or female gamete production, and the presence of flat leaf-like structures. In our male clone, the *LFY-*mutated trees often formed replicated floral structures, which was not a predicted outcome given that our field trial of natural flowering with RNAi targeting of *LFY* in poplar gave reduced catkin size and very underdeveloped flowers [5, 6]. However, we observed a similar re-iterative floral form in eucalypts with CRISPR targeting of the *LFY* homolog under floral acceleration with a constitutive promoter [10]. As also discussed in the eucalypt study, we believe that it is likely that continued exposure to *FT* leads to continued floral meristem activity and thus continued floral organ formation that would not occur under natural flowering.

Targeting of both *AG1* and *AG2* led to flowers with replicated floral structures in both our male and female trees, indicative of a loss of floral determinacy (Figures 4, 8). As *AG* genes are known to have a role in floral meristem determination, this phenotype fits what would be predicted for loss of *AG* function and aligns well with our field observations of RNAi targeting of *AG* genes in female poplar [7]. In our male *AG*-targeted trees, we observed an absence of anthers. In our female *AG*-targeted trees, we observed an absence of well-formed carpels. These data are consistent with a homeotic conversion of organ identity due to loss of *AG* function. For female clone 717, there was an absence of carpels in the CRISPR-targeted trees, which should lessen the ability of these trees to form ovules or wind-dispersible seeds. For male clone 353, there was an absence of anthers in the CRISPR-targeted trees, indicative of reduced potential for pollen formation, which would decrease the possibility of gene flow via pollen grains. Overall, our findings support that targeting of these key floral genes by CRISPR would dramatically reduce, if not eliminate, the ability of the trees to undergo sexual reproduction.

We observed striking variation in floral form, both within and between ramets and gene insertion events. Variable floral form may be of horticultural interest, as a single plant could exhibit great variation in floral form during the growing season under normal variation in temperature. This phenomenon has been observed in multiple species, with flowering differing within a single shoot, between shoots of a single plant, or between individuals in a single population [45]. This outcome speaks to the developmental plasticity of flowering onset and organ development due to the variation in insertion sites of *FT*, different degrees of juvenility (and thus competence to respond to FT induction) depending on plant size and meristem position [40], and the wide variation in heating regimes within and between plants in the greenhouse environment (with typical wide spatial variation in temperature and lighting). In addition, floral development is considered a highly polygenic trait, with expression dependent on many genes in addition to *FT* [46]. There may be hundreds of genes affecting flowering that may also have differential responses to high temperature microenvironments. Other laboratories have made similar observations; a similar gradient of floral form, ranging from well-developed to more vegetative types of flowers, has been observed in other studies of genetically accelerated poplar trees [47].

Chimerism for knockout mutations may be another cause of variation in floral form within trees and events. However, initial characterization of CRISPR lines used to select these particular events for re-transformation found a very low level of suspected chimerism and no evidence of off-target changes for a subset of CRISPR sites similar to those targeted [12]. A prior study of CRISPR targeting of the 4CL1 gene in poplar, where the phenotype can be assessed by a readily-observable wood color, showed no evidence of phenotype variation after 4 years of cutting back and re-growing shoots of selected transgenic events [48]. Therefore, it is unlikely that chimerism can account for a substantial amount of the variation in floral form that we observed.

We observed bi-sexual flowers in clone 353 (Supplemental Figure S5). These flowers were present on both *AtFT*-only flowering control and Cas9-only control trees. Such bisexual flowers on male clone 353 have been documented previously for genetically accelerated flowering trees in the greenhouse [40]. We did not observe bisexual or female flowers on CRISPR knockout *LFY* events in 353 as we previously observed in naturally flowering field-grown RNAi-*LFY* trees [44]. This difference is likely attributable to the system used (RNAi vs CRISPR), as RNAi may not eliminate all target gene expression like CRISPR is expected to do. However, to validate these results, and all the effects of CRISPR knockout on floral form and fertility reported above, it is essential to test trees under natural maturation and flowering in the field. Field studies would mitigate possible side effects of heat shock and would allow for flowering in the context of standard floral regulators. This work will begin once natural flowering of CRISPR knockout trees begin in a year or few.

## Conclusions

Heat-induced rapid flowering enabled us to observe strong effects on floral form and fertility from knockouts of the *LFY* and *AG* genes in poplar. The marked variability in floral form we observed shows the striking developmental plasticity of flowering onset and morphology and suggests possible new means to create floral diversity of horticultural value.

## Materials and methods

### Peptide predictions and alignments

The previously obtained allele sequence information [12] was used to obtain predicted peptides using an online translation tool (www.expasy.org/translate). Predicted *LFY* peptides were aligned to compare WT *LFY* alleles of both 717 and 353 with mutants in those clones. Peptide alignments were created using Clustal Omega [49].

### Vector construction, event selection, and plant transformation

To create the HSP:*AtFT* vector, the *FT* gene from *Arabidopsis thaliana* was cloned into the vector pCAMBIA under control of a heat-shock inducible promoter (HSP, see supplemental Figure S8). This vector was used to transform WT control and the selected Cas9 control and CRISPR events. Five *LFY* mutants were selected for re-transformation, two in clone 353 and three in clone 717 (See Supplemental Table S3). Four *AG* double mutants were selected for re-transformation, two in clone 353 and two in clone 717 (see Supplemental Table S4) These mutants were previously identified and characterized regarding their genetic changes in the target genes [12]. Mutants were selected for having predicted loss-of-function changes to both alleles of either *LFY* or both *AG1* and *AG2*. Cas9-only control trees were selected as having no changes in target genes. Allele-specific sequence information for these events can be found in Supplemental File S1. Transformation occurred using standard organogenic techniques with hygromycin selection [50]. A total of 130 *AtFT* sub-events in clone 353 and a total of 97 *AtFT* sub-events in clone 717 were selected for heat induction (see Supplemental Tables S5, S6). Re-transformed events were confirmed by genotyping with the primers HSP FT-F01 5′-AGTGAAGGCATCGTATCAAGC-3′ and HSP FT-R01 5′-CGCGGGATATCACCACTTTG-3′. Confirmed events were propagated by shoots to obtain multiple ramets per event. Small rooted ramets were transplanted to soil and grown in a greenhouse in the absence of purposeful heat induction, daytime temperature of 25C, nighttime temperature of 21C, with 16 hours of light and 8 hours of dark. Prior to heat induction, trees were trimmed then singled to retain one main shoot. Trees with a height of 40 cm or more were used for heat induction.

### Heat induction

Two greenhouses were used for growth and heat induction of selected ramets, these were designated greenhouse A and greenhouse B. Trees were randomized within each greenhouse such that each greenhouse was an experimental block. Heat-induction greenhouses included supplementary fans and heaters to aid in air circulation and quick heating. Plants were well watered prior to the start of daily heat induction. The daily heat induction goal was 43C from 11:00 a.m. to 5:00 p.m., with a 2-degree heat increase for cloudy days. At 5:00 p.m. the temperature was set to 29°C, this was reduced to 23°C at 10:00 p.m., and increased to 29°C after 6:00 a.m. A graph over average hourly temperatures per greenhouse and a view of one set of 717 trees prior to the onset of heat induction is shown in Supplemental Figure S9.

### Plant growth measurements, floral scoring, and floral imaging

Ramet size was measured as stem height (soil level to plant apex) using a meter stick. All ramets were screened for the presence or absence of catkins or catkin-like structures. Ramets with at least 1 catkin or catkin-like structure were scored as flowering. Events were scored as flowering if at least 1 ramet from that event was scored as flowering. Whole plants were photographed using a Canon Rebel XSI digital camera as were whole catkins. Detached catkins were imaged using a Keyence digital dissecting microscope.

## Supplementary Material

Web_Material_uhad132

## Data Availability

The data underlying this article are available in the article and in its online supplementary material.
