# Supplementary material for: Variation in floral form of CRISPR knock-outs of the poplar homologs of LEAFY and AGAMOUS after FT heat-induced early flowering
Source: Hortic Res. 2023 Jun 29;10(8):uhad132. doi: 10.1093/hr/uhad132 (PMC10410293; doi:10.1093/hr/uhad132)
Supplement: Web_Material_uhad132 [file web_material_uhad132.zip › supplemental figures and tables.pdf]

Starting construct	Re-transformation construct	# of sub-events obtained	# of flowering sub-events	% of flowering sub-events	# of ramets obtained	# of flowering ramets	% of flowering ramets
None (WT control)	None	NA	0	0	10	0	0
None (flowering control)	35S:AtFT	22	7	31.8	107	18	16.8
Cas9 event 34	35S:AtFT	3	0	0	15	0	0
Cas9 event 51	35S:AtFT	7	1	14.3	35	ND	ND
DL 143	35S:AtFT	23	12	52.2	110	27	24.5
DL 106	35S:AtFT	12	10	83.3	74	ND	ND

Supplemental Table 1: Inventory of male clone 353 that were re-transformed with the construct 35S:AtFT and grown in a standard greenhouse to allow for induced early flowering. ND is no data, ramet-specific floral data was not collected.

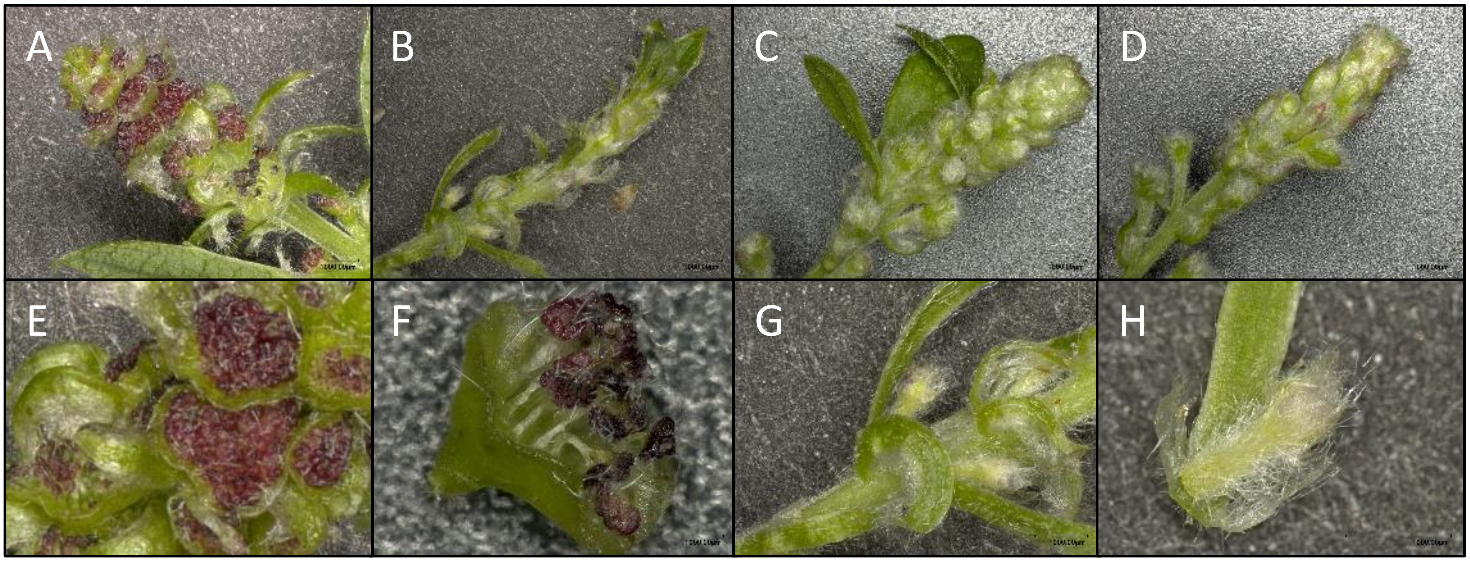

Supplemental Figure 1: Selected plants of male clone 353 were re-transformed with the construct 35S:AtFT and grown in a standard (non-heat induced) greenhouse to allow for induced early flowering
Examples of catkins obtained from (A) flowering control and (B-D) three different 35S:AtFT re-transformation events in DL 143 plants. Example of floral organ development from (E, F) flowering control and (G, H) DL event 143.

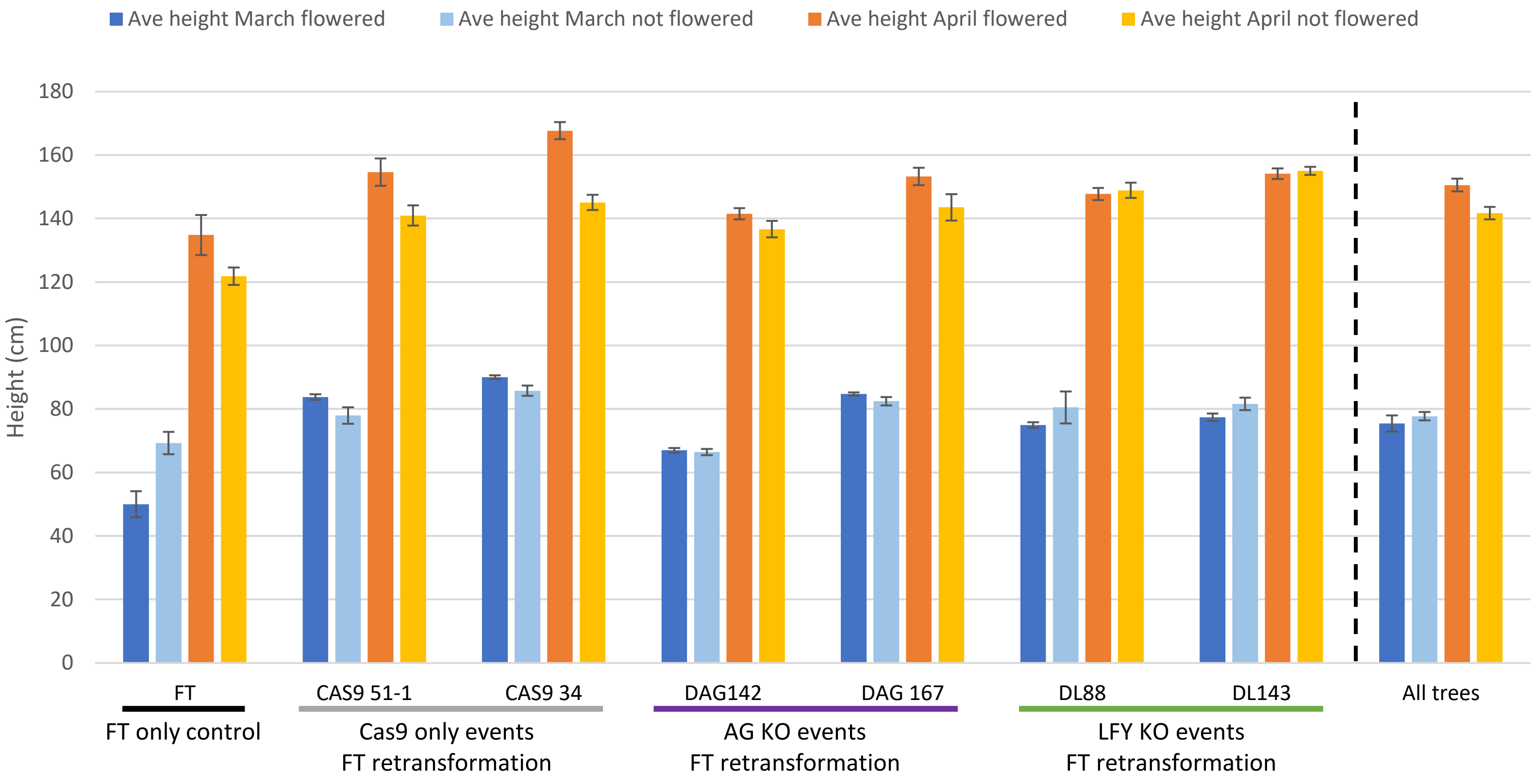

Supplemental Figure 2: Flowering and non-flowering 353 trees showed similar tree heights

Tree height of all trees was measured in both March and April for all living 353 trees. Bars show average heights of trees within an event, or across all trees. Standard error shown.

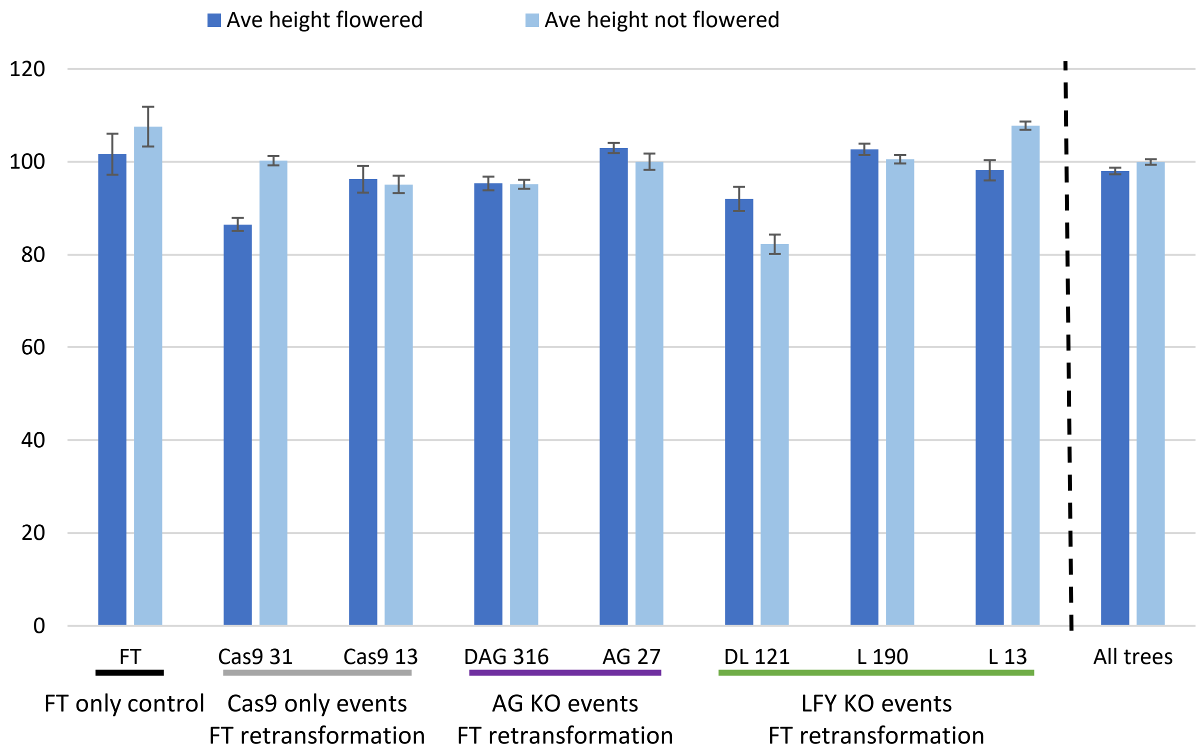

Supplemental Figure 3: Flowering and non-flowering 717 trees showed similar tree heights

Tree height of all trees was measured once for all living 717 trees. Bars show average heights of trees within an event, or across all trees. Standard error shown.

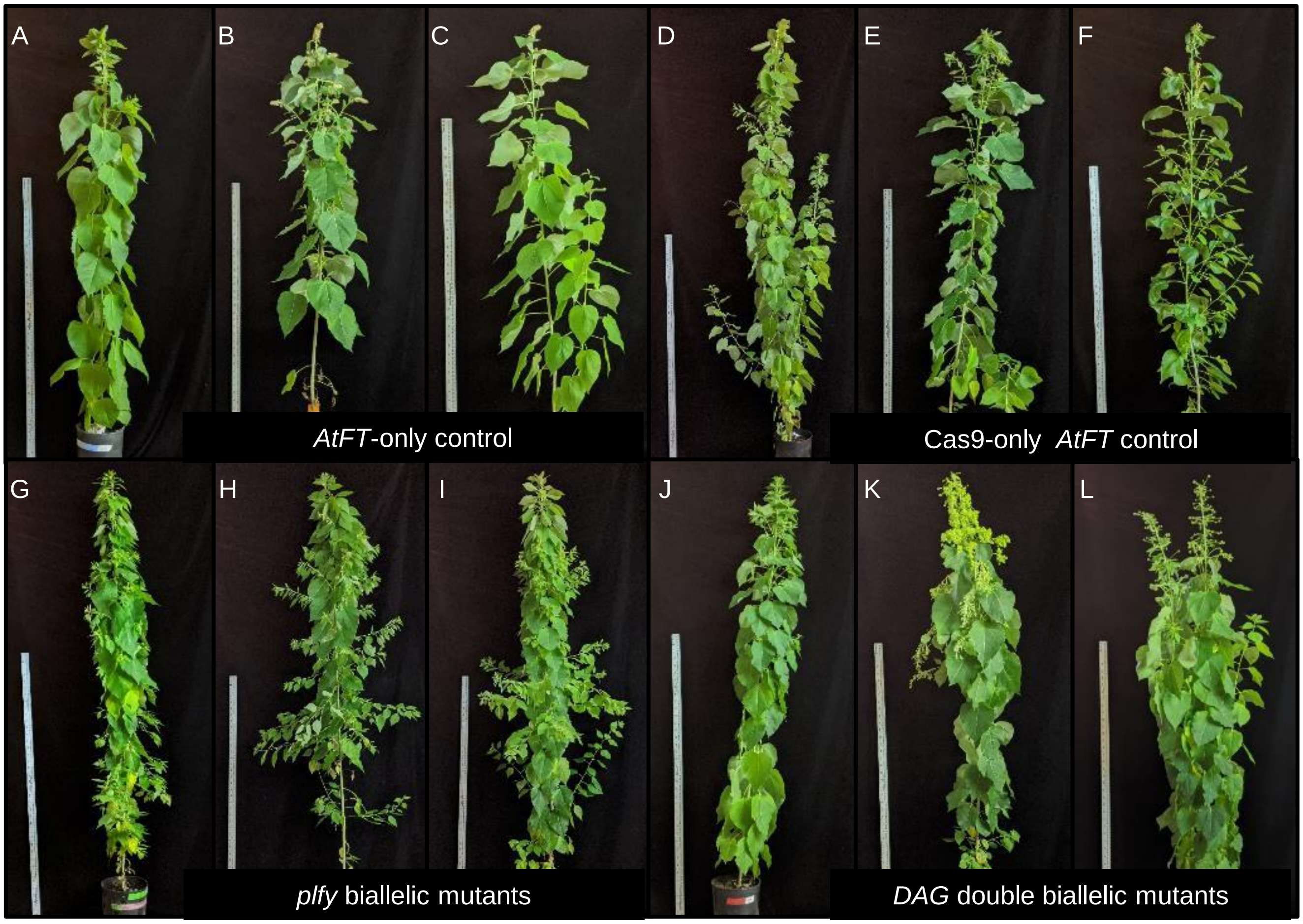

Supplemental Figure 4: Whole-tree images of male clone 353 after heat induction

(A-C) *AtFT*-only control trees, (D-F) three different Cas9-only *AtFT* event control trees, (G-I) three different *lfy AtFT* events, (J-L) three different DAG *AtFT* events.

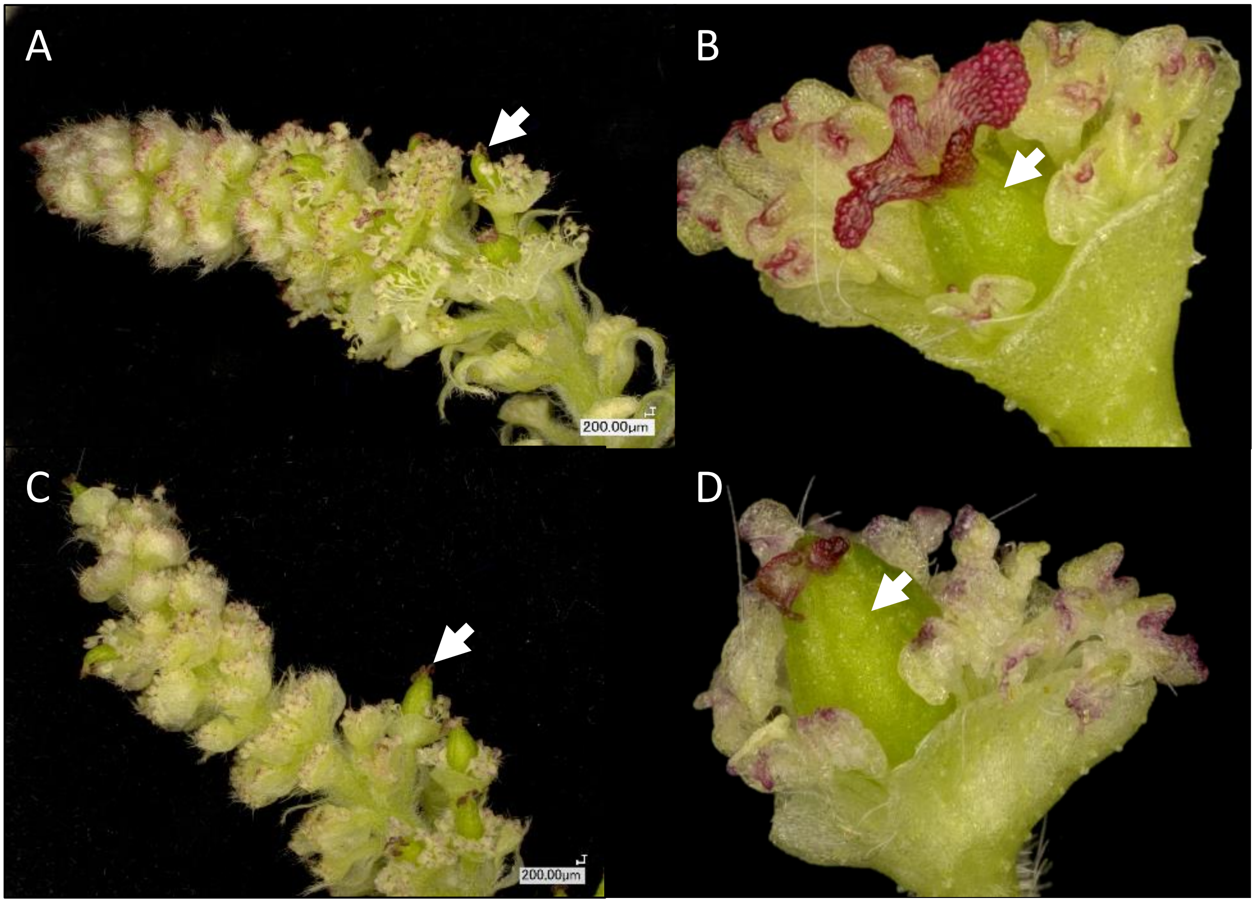

Supplemental Figure 5: Occasional carpels were observed on control trees in male clone 353 transformed with HSP:*AtFT*
These carpels were surrounded by anthers. This outcome was observed in (A, B) *AtFT*-only control event 42 ramet 6 as well as (C, D) Cas9-only *AtFT* event 45 ramet 5. Selected carpels shown with white arrows.

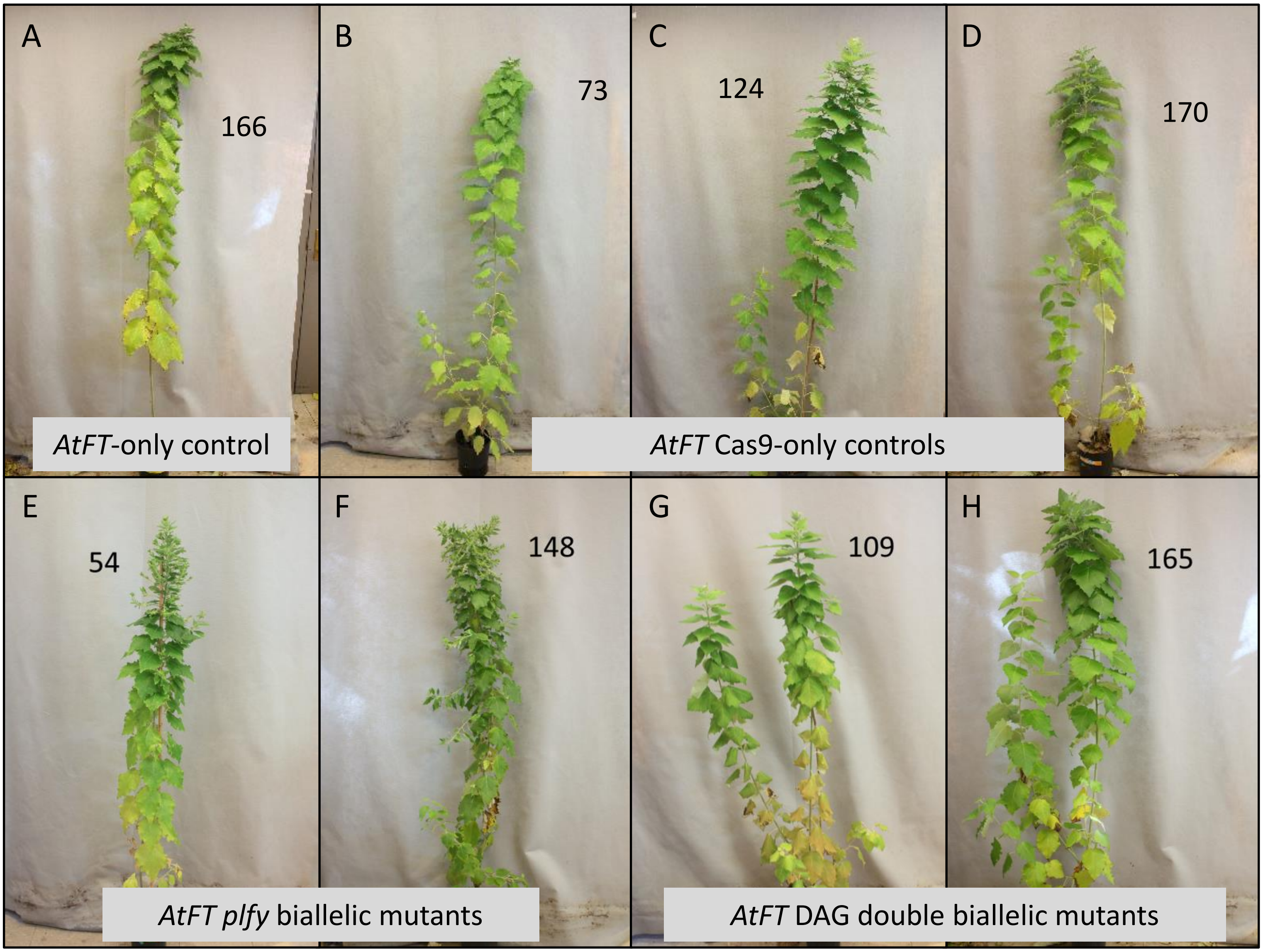

Supplemental Figure 6: Whole plant images of female clone 717 after heat induction

(A) *AtFT*-only control, (B-D) Cas9-only *AtFT* control, (E, F) *plfy* biallelic mutants, (G, H) *pag1 pag2* double biallelic mutants, Numbers by trees indicate randomization number. Note abnormal upper crowns only in the *lfy* mutants.

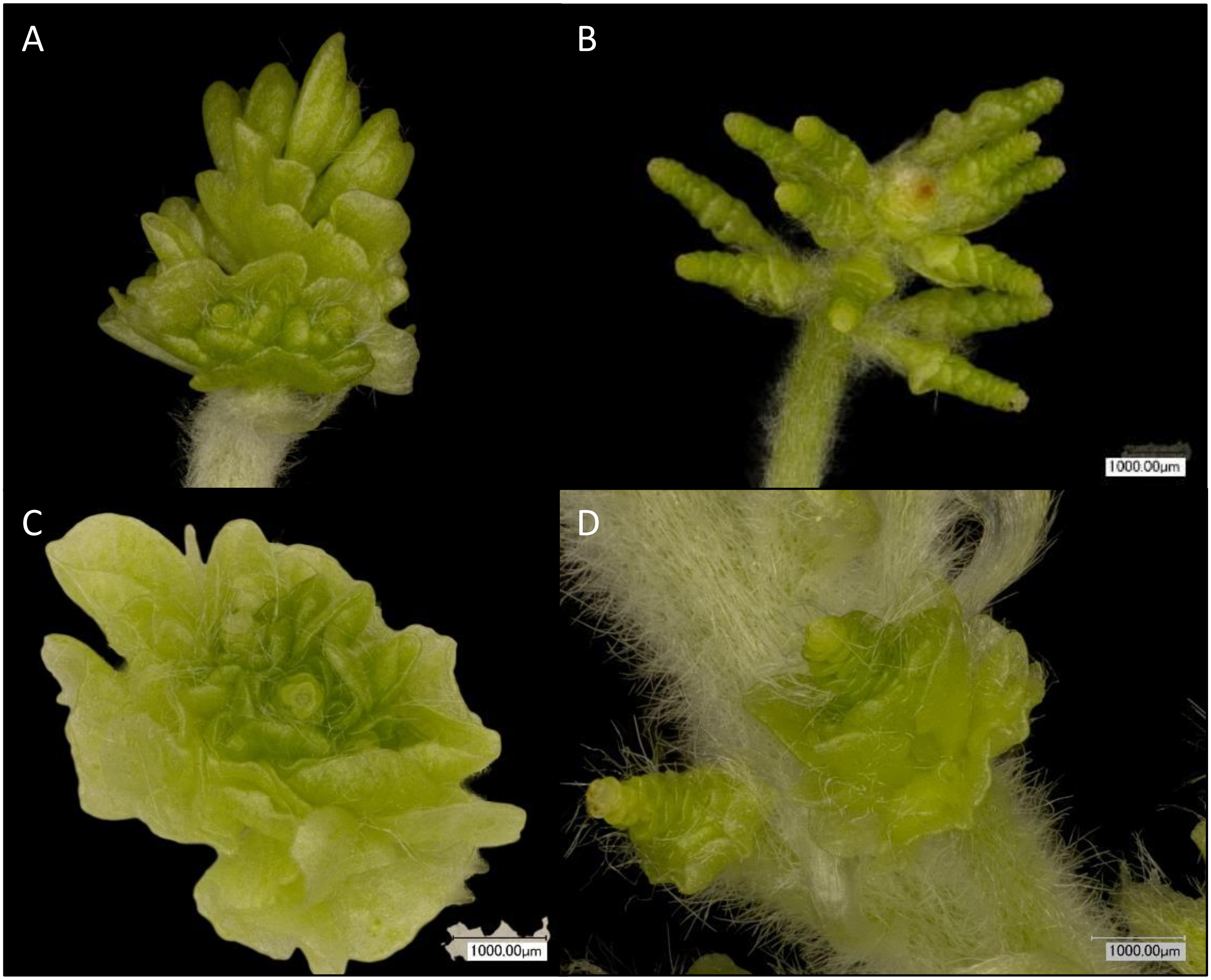

Supplemental Figure 7: Examples of intra-tree, intra-sub-event, and intra-event similarities and variation among 717 AG knock-out flowers

Flowers of DAG event 27 *AtFT* sub-event 44 from (A) tree 109 and (B) tree 45; these trees are clonal replicates. (C) Flower of DAG event 27 *AtFT*-sub-event 70 tree 18. This tree is the same DAG event as panels A and B but in a different *AtFT* sub-event. (D) Flowers of DAG event 316, *AtFT* sub-event 23, tree 50. This is a different DAG knockout event from the other three panels and a different *AtFT* sub-event.

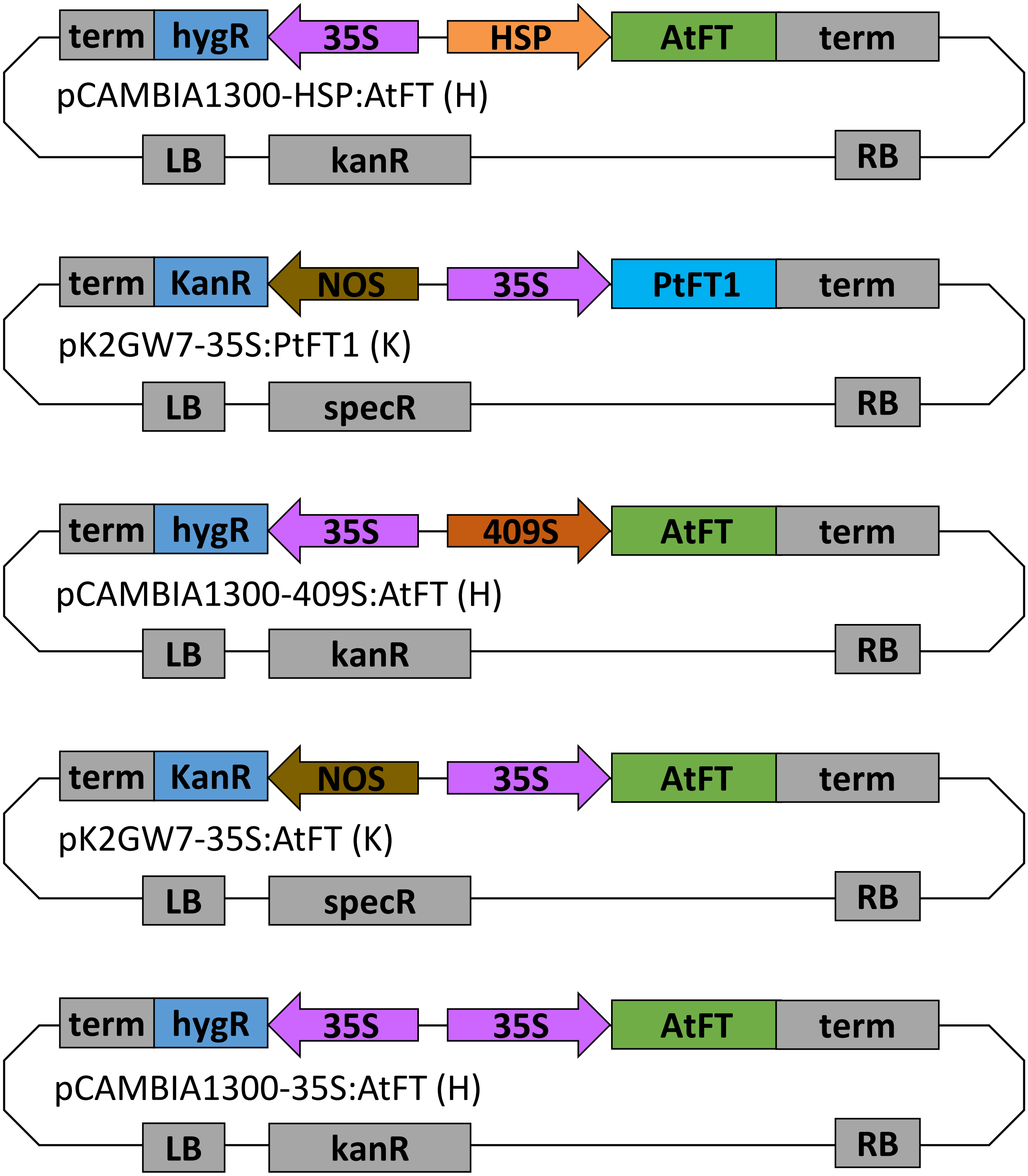

Supplemental Figure 8: Constructs used for transformation

A series of constructs were created in the pCAMBIA1300 and pK2GW7 vectors to express either the *FT* gene from *Arabidopsis thaliana* (*AtFT*) or the *FT1* gene from *Populus trichocarpa* (*PtFT1*) under control of different promoters. These included a heat-inducible promoter (HSP), the constitutive cauliflower mosaic virus promoter (35S), or the constitutive 409S promoter (409S). Vectors contained either the hygromycin (hygR) or kanamycin (KanR) selectable marker for use in plants as well as either the kanamycin (KanR) or spectinomycin (specR) selectable marker for use in bacteria. Term is terminator, LB and RB are the T-DNA right border and left border, respectively. Arrows indicate promoter directionality.

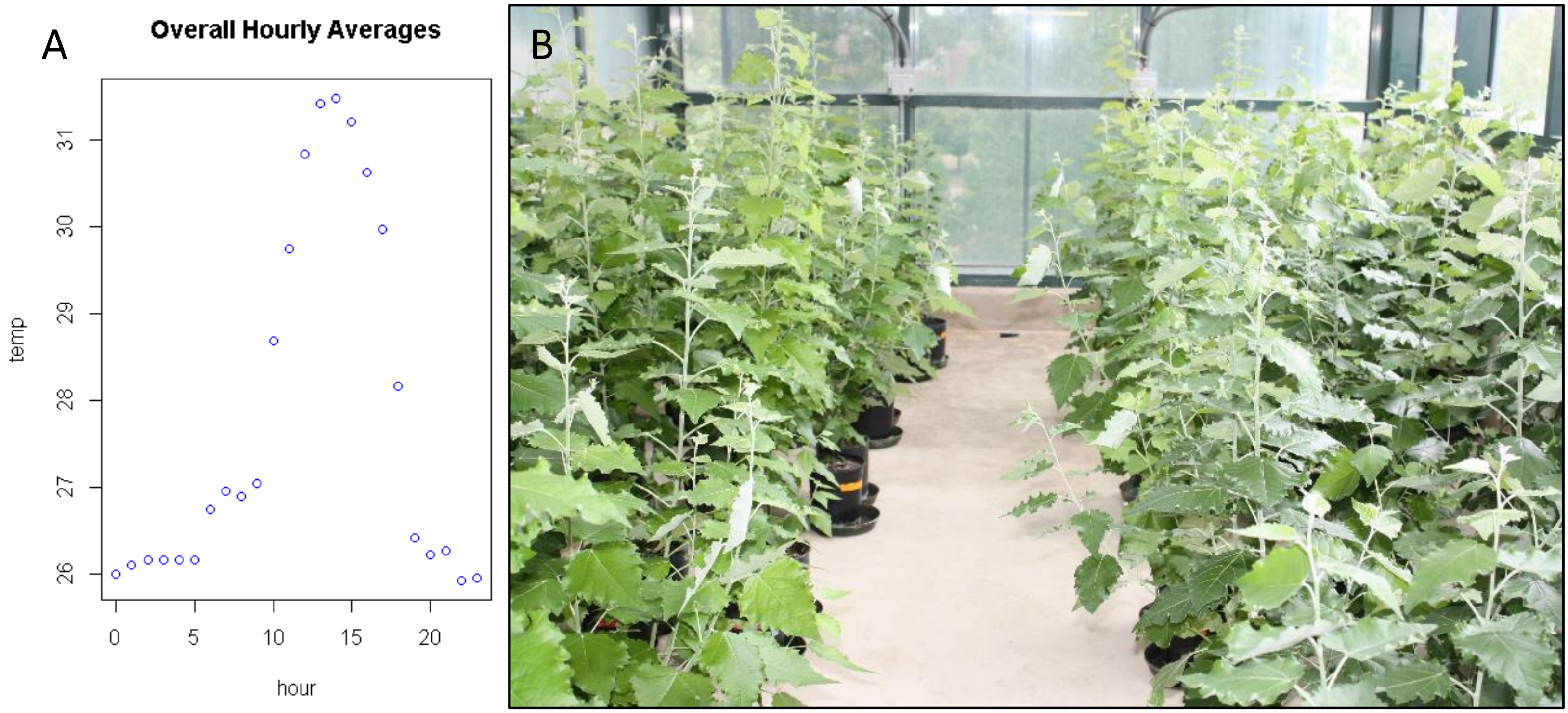

Supplemental Figure 9: Heat induction pattern and view of the heat-induction greenhouse
(A) An average heat induction cycle included a basal temperature of 26 degrees, followed by a peak of 32-degree heat induction. (B) Male clone 717 trees in a heat-induction greenhouse prior to the onset of the heat-induction.

Clone	Construct	# total events	# flowering events	% flowering events
**717 *P. tremula* x *P. alba***	HSP:AtFT (K)	29	9	31.0
**353 *P. tremula* x *P. tremuloides***	HSP:AtFT (K)	33	16	48.5
**717 *P. tremula* x *P. alba***	35S:PtFT1 (K)	8	0	0.0
**353 *P. tremula* x *P. tremuloides***	35S:PtFT1 (K)	23	2	8.7
**717 *P. tremula* x *P. alba***	409S:AtFT (H)	32	1	3.1
**353 *P. tremula* x *P. tremuloides***	409S:AtFT (H)	26	7	26.9
**Brauna *P. tremula***	35S:AtFT (K)	8	0*	0.0
**T89 *P. tremula* x *P. tremuloides***	35S:AtFT (K)	3	0*	0.0

Supplemental Table 2: Inventory of early-flowering constructs tested in different poplar clones

HSP is a heat-shock-inducible promoter, 35S is a strong constitutive viral promoter, 409S is a moderate constitutive promoter, *AtFT* is from *Arabidopsis thaliana*, *PtFT1* is from *P. trichocarpa,* (H) refers to hygromycin selection in plants, (K) refers to kanamycin selection in plants. Events were scored as flowering if at least 1 ramet from that event flowered in greenhouse conditions. * Indicates flowering was observed in tissue culture.

LFY mutants selected for retransformation			
**clone**	**event**	**Allele 1**	**Allele 2**
717	L1C 13	1 bp deletion	1 bp deletion
717	L1C 190	1 bp deletion	1 bp deletion
717	DL 121	120 bp deletion	120 bp deletion
353	DL 88	120 bp deletion	120 bp deletion
353	DL 143	120 bp deletion	120 bp deletion

Supplemental Table 3: *LFY* mutants selected for re-transformation
Five independent *lfy* mutants were selected for re-transformation with HSP-*AtFT*, three in female clone 717 and two in male clone 353. Event names are from initial mutant identification and analysis. Both *LFY* alleles were characterized in each event, bp = base pair.

AG1/AG2 mutants selected for retransformation					
		AG1		***AG2***	
**clone**	**event**	**Allele 1**	**Allele 2**	**Allele 1**	**Allele 2**
717	AG2C 27	491 bp insertion	1 bp insertion	1 bp deletion	1 bp deletion
717	DAG 316	41 bp deletion	41 bp deletion	4 bp deletion	5 bp deletion
353	DAG 142	41 bp deletion	2 bp deletion	1 bp insertion, 3 bp deletion	2 bp deletion
353	DAG 167	5 bp deletion	4 bp deletion	1 bp deletion, 5 bp deletion	1 bp deletion

Supplemental Table 4: *AG* mutants selected for re-transformation

Four independent *AG1/AG2* double mutants were selected for re-transformation with HSP-*AtFT*, two in female clone 717 and two in male clone 353. Event names are from initial mutant identification and analysis. Both alleles of each *AG1* and *AG2* were characterized, bp = base pair.

poplar clone	Type of mutant	Construct	Event	FT subevents obtained	FT subevents for GH study	Total ramets
353	KO	LFY1/3C	88	47	25	50
353	KO	LFY1/3C	143	46	25	50
353	Cas9	Cas9	34	47	10	20
353	Cas9	Cas9	51-1	24	10	20
353	KO	AG1/2C	142	48	25	50
353	KO	AG1/2C	167	45	25	50
353	NA	HSP::AtFT	NA	1	1	10
**Total 353 events**				**257**	**121**	**250**

Supplemental Table 5: Selection of 353 events for greenhouse study
Plants were randomly arranged in two blocks (two greenhouse rooms A & B) with 1 ramet per sub-event per block.

Poplar clone	Type of mutant	Construct	Event #	FT subevents obtained	FT subevents For GH study	Total ramets
717	KO	LFY1C	13	45	14	28
717	KO	LFY1C	190	35	14, 17	31
717	KO	LFY1/3C	121	32	4	8
717	KO	AG2C	27	39	15	30
717	KO	AG1/2C	316	26	19	38
717	Cas9	Cas9	13	6	4	8
717	Cas9	Cas9	31	10	9	18
717	none	HSP::AtFT	142-1	1	1	9
**Total 717 events**				**193**	**97**	**170**

Supplemental Table 6: Selection of 717 events for greenhouse study

Plants were randomly arranged in two blocks (two greenhouse rooms A & B) with 1 ramet per sub-event per block.

	GH A		GH B		total	total
**status**	**#**	**Percentage**	**#**	**Percentage**	**#**	**Percentage**
Flowering	29	23.4%	27	21.4%	56	22.4%
Not flowering	94	75.8%	96	76.2%	190	76.0%
Dead	1	0.8%	3	2.4%	4	1.6%
Total	124		126		250	100%

Supplemental Table 7: Flowering rates for clone 353
GH A and GH B indicates which greenhouse (A or B) the trees were placed in for heat-induction. Rates are percentage of tested trees that had any flowering.

	GH A		GH B		total	total
**Status**	**#**	**Percentage**	**#**	**Percentage**	**#**	**Percentage**
Flowering	43	49.4%	15	18.1%	58	34.1%
Not flowering	40	46.0%	67	80.7%	107	62.9%
Dead	4	4.6%	1	1.2%	5	2.9%
Total	87		83		170	100%

Supplemental Table 8: Flowering rates for clone 717
GH A and GH B indicates which greenhouse (A or B) the trees were placed in for heat-induction. Rates are percentage of tested trees that had any flowering.
