# Supplementary material for: Variation in floral form of CRISPR knock-outs of the poplar homologs of LEAFY and AGAMOUS after FT heat-induced early flowering
Source: Hortic Res. 2023 Jun 29;10(8):uhad132. doi: 10.1093/hr/uhad132 (PMC10410293; doi:10.1093/hr/uhad132)
Supplement: Web_Material_uhad132 [file web_material_uhad132.zip › supplemental file 1 all allele sequence data v2.docx]

**Gene and allele sequence information**

Target sites, start codons, and SNPs are underlined and in bold. Key to SNPs follow the standard code of K, G or T; M, A or C; R, A or G; Y, C or T; W, A or T.

**Male clone 353**

LFY alleles

>WT_LFY_353

CTGTCCAGTTCCGAAGAAACATCAAAACCCTTTAATTCTGTTAGCTTCCCAATACATACAAAAAAGAAAAAAAGACAA**R**AAACTTGTCCTGTTAAGGGCAGTTTTGGTATA**Y**AAATAAAACAAGAAGCTCACTTGTCTTTATATATCTACCAAATCCAAGACATGCACCAGTGAAA**GATCACAGAGAGAGAGACAA**GGGGGCAGATAGAT**ATG**GATCCGGAGGCTTTCACGGCGAGTTTGTTCAAATGGGA**Y**ACGAGAGCAATGGTGCCACATCCTAACCG**Y**CTGCTTGAAATGGT**GCCCCCGCCTCAGCAGCCAC**CGGCTGCGGCGTTTGCTGTAAGGCCAAGGGAGCTATGTGGGCTAGAGGAGTTGTTTCAAGCTTATGGTATTAGGTACTACACGGCAGCGAAAATAGCTGAACTCGGGTTCACAGTGAACACCCTTTTGGACATGAAAGATGAGGAGCTTGATGAAATGATGAATAGTTTGTCTCAGATCTT**Y**AGGTGGGATCTTCTTGTTGGTGAGAGGTATGG

>22266_15_L1L3c88F_LFY_seq_F7

NNNNNNNNNTGNNTAGTTCGATTTCACTGTCCAGTTCCGAAGAAACATCAAAACCCTTTAATTCTGTTAGCTTCCCAATACATACAAAAAAGAAAAAAAGACAAAAAACTTGTCCTGTTAAGGGCAGTTTTGGTATACAAATAAAACAAGAAGCTCACTTGTCTTTATATATCTACCAAATCCAAGACATGCACCAGTGAAAGATCACAGAGAGAGAGACACCGGCTGCGGCGTTTGCTGTAAGGCCAAGGGAGCTATGTGGGCTAGAGGAGTTGTTTCAAGCTTATGGTATTAGGTACTACACGGCAGCAAAAATAGCTGAACTCGGGTTCACAGTGAACACCCTTTTGGACATGAAAGATGAGGAGCTTGATGAAATGATGAATAGTTTGTCTCAGATCTTCAGGTGGGATCTTCTTGTTGGTGAGAGGTATGGTATTAAA

>22731_11_143F_LFY_seq_F7

NNNNNNNNNNGNNAANNNTTGCNNANNTTCACTGTCCAGTTCCGAAGAAACATCAAAACCCTTTAATTCTGTTAGCTTCCCAATACATACAAAAAAGAAAAAAAGACAAAAAACTTGTCCTGTTAAGGGCAGTTTTGGTATACAAATAAAACAAGAAGCTCACTTGTCTTTATATATCTACCAAATCCAAGACATGCACCAGTGAAAGATCACAGAGAGAGAGACACCGGCTGCGGCGTTTGCTGTAAGGCCAAGGGAGCTATGTGGGCTAGAGGAGTTGTTTCAAGCTTATGGTATTAGGTACTACACGGCAGCAAAAATAGCTGAACTCGGGTTCACAGTGAACACCCTTTTGGACATGAAAGATGAGGAGCTTGATGAAATGATGAATAGTTTGTCTCAGATCTTCAGGTGGGATCTTCTTGTTGGTGAGAGGTATGG

AG alleles

>WT_AG1_353

GCAGCT**ATG**GAATATCAAAATGAATCCCTTGAGAGCTCCCC**M**CTGAGGAAGCTGGGAA**GGGGAAAGGTGGAGATCAAG**CGGATCGAGAACACCACCAATC**GCCAAGTCACTTTCTGCAAA**AGGCGCAGTGGTTTGCTCAAGAAAGCCTACGAATTATCTGTTCTTTGCGATGCTGAGGTTGCACTCATCGTCTTCTCTACCCGCGGTCGCCTTTATGAGTACTCTAACGATAGGTAAATAAATCTAATTTTAGATATATGCTTCTCTGGATCTTAAATTCTCC

>23039_03_142A_AG1_seq_R4

NNNNNNNNNNNCANNNNNTNNNNNATNCGTNCGAAGTTACTNCATTCANGGGCGACCGCGGGTAGAGAANNGATGAGTGCAACCTCAGCATCGCAAAGAACAGATAATTCGTAGGCTTTCTTGAGCAAACCACTGCGCCTTTTGGATCTCCACCTTTCCCCTTCCCAGCTTCCTCAGTGGGGAGCTCTCAAGGGA

>23039_06_167A_AG1_seq_R4

NNNNNNNNNNNNTCNNNTNTTTTNANCTTNTTCGTTTNGAAGTTCNTCATTNAAGGCGACCGCGGGTAGAGAAGACGATGAGTGCAACCTCAGCATCGCAAAGAACAGATAATTCGTAGGCTTTCTTGAGCAAACCACTGCGCCTTTTGCAGAAAGTGACTTGGCGATTGGTGGTGTTCTCGATCCGCTTCCACCTTTCCCCTTCCCAGCTTCCTCAGTGGGGAGCTCTCAAGGGA

RC

TCCCTTGAGAGCTCCCCACTGAGGAAGCTGGGAAGGGGAAAGGTGGAAGCGGATCGAGAACACCACCAATCGCCAAGTCACTTTCTGCAAAAGGCGCAGTGGTTTGCTCAAGAAAGCCTACGAATTATCTGTTCTTTGCGATGCTGAGGTTGCACTCATCGTCTTCTCTACCCGCGGTCGCCTTNAATGANGAACTTCNAAACGAANAAGNTNAAAANANNNGANNNNNNNNNNNN

>23039_22_167B2_AG1_seq_R4

ANCNNNNNNNGNCTNATNNAAGCATTGTCCGATAATNATCAGGCGACGCGGGTCGAGAAACGATGAGTGCAACCTCAGCATCGCAAAGAACAGATAATTCGTAGGCTTTCTTGAGCAAACCACTGCGCCTTTTGCAGAAAGTGACTTGGCGATTGGTGGTGTTCTCGATCCGCTTGCCACCTTTCCCCTTCCCAGCTTCCTCAGGGGGGAGNCTCTCAAGGGA

RC

TCCCTTGAGAGNCTCCCCCCTGAGGAAGCTGGGAAGGGGAAAGGTGGCAAGCGGATCGAGAACACCACCAATCGCCAAGTCACTTTCTGCAAAAGGCGCAGTGGTTTGCTCAAGAAAGCCTACGAATTATCTGTTCTTTGCGATGCTGAGGTTGCACTCATCGTTTCTCGACCCGCGTCGCCTGATNATTATCGGACAATGCTTNNATNAGNCNNNNNNNGNT

>WT_AG2_353

**ATG**GCATACCAAAATGAATCCCAAGAGAGCTCTCCCCTGAGGAAGCTGGGGA**GGGGAAAGGTGGAGATCAAG**CGGATCGAGAACACCAC**M**AATC**GCCAAGTCACTTTCTGCAAA**AGGCGGAATGGTTTGCTCAAGAAAGCCTATGAATTATCTGTTCTTTGCGATGCTGAGGTTGCACTCATCGTCTTCTCCAGCCGTGGACGCCTTTATGAGTACTCTAACAATAGGTATAT**R**CTTAGTTCCT**Y**G**K**CTCATGAATTCTCCATGTTGCAAGCCCTCTTCAAGTGCTCACAGTTGGTTTTTCTTGCTTTCTCATCCAAAGGGATTTG**W**TTTTTCTTTTTGTTTATGCCAGGGTTAATTTTTATGGTTTTT

>23139_01_142A_AG2_seq_F1

NNNNNNNNNNCNNNGCAGCAGCTATGGCANACCAAAATGAATCCCAAGAAGAAGCTCTCCCCTGAGGAAGCTGGGGAGGGGAAAGGTGGAGATCCCGGATCATCAACAACACCACTCATCGCCTCATTTTTTTCAAAAGGCGGAATGGTTTGCTCAAGAAAGCCTATGAATTATCTGTTCTTTGCGATGCTGAGGTTGCACTCATCGTCTTCTCCAGCCGTGGACGCCTTTATGAGTACTCTAACAATAGGTATATGCTTAGTTCCTCGGCTCATGAATTCCNCATGTTGCAAAA

>23139_04_167A_AG2_seq_F1

NNNNNNCNNCTGGCAGCAGCTATGGCATACCAAAATGAATCCCAAGAGAGCTCTCCCCTGAGGAAGCTGGGGAGGGGAAAGGTGGAGACAAGCGGATCGAGAACACCACCAATCGCCAAGTCACTTTCTGCGAAAGGTGGATTGGTTTGCACAACAAAGCCTATAACTTATCTTTGCTTTGCTAAGGTTACGTTCCTCTCCTCCTCTTCCCCAGGACGGCATTATTATATCACTACCCTAACGATAAGGCTATACTCCTTTCCTCGGGACATGAATTCGCNGCAATGCAAN

**Female clone 717**

LFY alleles

>WT_LFY_717

CTGTCCAGTTCCGAAGAAACATCAAAACCCTTTAATTCTGTTAGCTTCC**Y**AATACATACAAAAAAGAAAAAAAGACAA**R**AAACTTGTCCTGTTAAGGGCAGTTTTGGTATA**Y**AAATAAAACAAGAAGCTCACTTGTCTTTATATATCTACCAAATCCAAGACATGCACCAGTGAAA**GATCACAGAGAGAGAGACAA**GGGGGCAGATAGAT**ATG**GATCCGGAGGCTTTCACGGCGAGTTTGTTCAAATGGGACACGAGAGCAATGGTGCCACATCCTAACCGTCTGCTTGAAATGGT**GCCCCCGCCTCAGCAGCCAC**CGGCTGCGGCGTTTGCTGTAAGGCCAAGGGAGCTATGTGGGCTAGAGGAGTTGTTTCAAGCTTATGGTATTAGGTACTACACGGCAGCGAAAATAGCTGAACTCGGGTTCACAGTGAACACCCTTTTGGACATGAAAGA**Y**GAGGAGCTTGATGAAATGATGAATAGTTTGTCTCAGATCTTTAGGTGGGATCTTCTTGTTGGTGAGAGGTATGGTATTAAAGCTGCTGTTAGAGCTGAAAGAAGAAGGCTTGATGAGGAGGATCCTAGGCGTAGGCAATTGCTCTCTGGTGATAATAATACAAATACTCTTGATGCTCTCTCCCAAGAAGGTTTGGTTAGCATTGATTCTACCTTTTAGTGTAATTAAGCTAAGCTCATACTATTACTAGCTATAGGAG**K**CCATGGCCGTTTT**R**

>20185_23_L1L3121_LFY_seq_F1

NNNNNNNNNNNNNNCTCACTTGTCTTTATATATCTACCAAATCCAAGACATGCACCAGTGAAAGATCACAGAGAGAGAGACACCGGCTGCGGCGTTTGCTGTAAGGCCAAGGGAGCTATGTGGGCTAGAGGAGTTGTTTCAAGCTTATGGTATTAGGTACTACACGGCAGCGAAAATAGCTGAACTCGGGTTCACAGTGAACACCCTTTTGGACATGAAAGACGAGGAGCTTGATGAAATGATGAATAGTTTGTCTCAGATCTTTAGGTGGGATCTTCTTGTTGGTGAGAGGTATGGTATTAAAGCTGCTGTTAGAGCTGAAAGAAGAAGGCTTGATGAGGAGGATCCTAGGCGTAGGCAATTGCTCTCTGGTGATAATAATACAAATACTCTTGATGCTCTCTCCCAAGAATGTTAGTATC

These 6 are all from 717 L1C 13

>21122_A01_13_8_M13R

NNNNNNCNNNNACCCTCACTAAAGGGACTAGTCCTGCAGGTTTAAACGAATTCGCCCTTAGCTGGCAAGTAACAACTTATAATAAGCTAATTATCTTATCCAAGTATACATAGAATTAATTTACTCTATAAAACTACAACCAAAAGGGAAATACAAAACGGCCATGGACTCCTATAGCTAGTAATAGTATGAGCTTAGCTTAATTACACTAAAAGGTAGAATCAATGCTAACCAAACCTTCTTGGGAGAGAGCATCAAGAGTATTTGTATTATTATCACCAGAGAGCAATTGCCTACGCCTAGGATCCTCCTCATCAAGCCTTCTTCTTTCAGCTCTAACAGCAGCTTTAATACCATACCTCTCACCAACAAGAAGATCCCACCTAAAGATCTGAGACAAACTATTCATCATTTCATCAAGCTCCTCATCTTTCATGTCCAAAAGGGTGTTCACTGTGAACCCGAGTTCAGCTATTTTCGCTGCCGTGTAGTACCTAATACCATAAGCTTGAAACAACTCCTCTAGCCCACATAGCTCCCTTGACCTTACAGCAAACGCCGCAGCCGGTGCTGCTGAGGCGGGGGCACCATTTCAAGCAGACGGTTAGGATGTGGCACCATTGCTCTCGTGTCCCATTTGAACAAACTCGCCGTGAAAGCCTCCGGATCCATATCTATCTGCCCCCTTGTCTCTCTCTCTGTGATCTTTCACTGGTGCATGTCTTGGATTTGGTAGATATATAAAGACAAGTGAGCTTCTTGTTTTATTTGTATACCAAAACTGCCCTTAACAGGAAGGGCGAATTCGCGGCCGCTAAATTCAATTCGCCCTATAGTGAGTCGTATTACAATTCACTGGCCGTCGTTTTACAACGTCGTGACTGGGAAAACCCTGGNGTTACCCAACTTAATCGCCTTGCAGCACATCCCCCTTTCGCCAGCTGGCGTANAGCGANAGGCCCGCNNNATCGCCCNNCCCANAGTGCNCANCTATACGTACGNAGTTTNNNTTACNCTANAAANNANANANCGTATCGNCTGNTTGNGNATGNNCNANNGANNNTNNTGANNCCNNNNNNCNACGGANGNGNATCCCCNNNNNGNNNNNNNNNGNNNNNNNATAANNNNTNCNGTNNNNNNNNNNNNNANNNNNNNNNNGCNNNNNNNNNNNNNTNNNNCNNNNNNCNNNNNNCCCNN

>21122_B01_13_10_M13R

NNNNNNANNNNACCCTCACTAAGGGACTAGTCCTGCAGGTTTAAACGAATTCGCCCTTAGCTGGCAAGTAACAACTTATAATAAGCTAATTATCTTATCCAAGTATACATAGAATTAATTTACTCTATAAAACTACAACCAAAAGGGAAATACAAAACGGCCATGGACTCCTATAGCTAGTAATAGTATGAGCTTAGCTTAATTACACTAAAAGGTAGAATCAATGCTAACCAAACCTTCTTGGGAGAGAGCATCAAGAGTATTTGTATTATTATCACCAGAGAGCAATTGCCTACGCCTAGGATCCTCCTCATCAAGCCTTCTTCTTTCAGCTCTAACAGCAGCTTTAATACCATACCTCTCACCAACAAGAAGATCCCACCTAAAGATCTGAGACAAACTATTCATCATTTCATCAAGCTCCTCATCTTTCATGTCCAAAAGGGTGTTCACTGTGAACCCGAGTTCAGCTATTTTCGCTGCCGTGTAGTACCTAATACCATAAGCTTGAAACAACTCCTCTAGCCCACATAGCTCCCTTGACCTTACAGCAAACGCCGCAGCCGGTGCTGCTGAGGCGGGGGCACCATTTCAAGCAGACGGTTAGGATGTGGCACCATTGCTCTCGTGTCCCATTTGAACAAACTCGCCGTGAAAGCCTCCGGATCCATATCTATCTGCCCCCTTGTCTCTCTCTCTGTGATCTTTCACTGGTGCATGTCTTGGATTTGGTAGATATATAAAGACAAGTGAGCTTCTTGTNNATTTGTATACCAAAACTGCCCTTAACAGGAAGGGCGAATTCGCGGCCGCTAAATTCAATTCGCCCTATAGTGAGTCGTATTACAATTCACTGGCCGTCGTTTTACAACGTCGTGACTGGGAAAACCCTGGNGTTACCCAACTTAATCGCCTTGCAGCACATCCCCCTTTCGCCAGCTGGCGTATAGCGANAGGCCCGCACNATCGCCCTTCCCANCAGTTGCGCANCTATACGTACGCAGTTTNNGTTTACNCNATNAAAGAGANANCNTATCGTCTNGTTTGNGNATGTACNGAGTGANNTNNTGANNNCNNNGGNCNAANGNANGNTGNATCCCCNNNNNNNNGNANGNCTGNNNGTCNNATAANNNNTNNNNNNANNNNNNNNGNNNNTNNNNNANNNNNNNNNGATGACNCNNNNNNNNNACGTGNN

>21122_C01_13_11_M13R

NNNNNNNNNNACCCTCACTAAAGGGACTAGTCCTGCAGGTTTAAACGAATTCGCCCTTAGCTGGCAAGTAACAACTTATAATAAGCTAATTATCTTATCCAAGTATACATAGAATTAATTTACTCTATAAAACTACAACCAAAAGGGAAATATAAAACGGCCATGGCCTCCTATAGCTAGTAATAGTATGAGCTTAGCTTAATTACACTAAAAGGTAGAATCAATGCTAACCAAACCTTCTTGGGAGAGAGCATCAAGAGTATTTGTATTATTATCACCAGAGAGCAATCGCCTACGCCTAGGATCCTCCTCATCAAGCCTTCTTCTTTCAGCTCTAACAGCAGCTTTAATACCATACCTCTCACCAACAAGAAGATCCCACCTAAAGATCTGAGACAAACTATTCATCATTTCATCAAGCTCCTCGTCTTTCATGTCCAAAAGGGTGTTCACTGTGAACCCGAGTTCAGCTATTTTCGCTGCCGTGTAGTACCTAATACCATAAGCTTGAAACAACTCCTCTAGCCCACATAGCTCCCTTGGCCTTACAGCAAACGCCGCAGCCGGTGCTGCTGAGGCGGGGGCACCATTTCAAGCAGACGGTCAGGATGTGGCACCATTGCTCTCGTGTCCCATTTGAACAAACTCGCCGTGAAAGCCTCCGGATCCATATCTATCTGCCCCCTTGTCTCTCTCTCTGTGATCTTTCACTGGTGCATGTCTTGGATTTGGTAGATATATAAAGACAAGTGAGCTTCTTGTTTTATTTATATACCAAAACTGCCCTTAACAGGAAGGGCGAATTCGCGGCCGCTAAATTCAATTCGCCCTATAGTGAGTCGTATTACAATTCACTGGCCGTCGTTTTACAACGTCGTGACTGGGAAAACCCTGGNGTTACCCAACTTAATCGCCTTGCAGCACATCCCCCTTTTCGCCAGCTGGCGTANAGCGANANNCCGCACCGATCGCCCTTCCCACAGTTGCGCANCTATACGTACGCAGTTNAGNTTACNCTATNAAAGANANNNNNTNTCGNCTGNTTGNGATGTANNAGTGANNNTNNGACACGCNNGGCNACNGATGGTGAATCCCCNNNNNAGTGNNCNNNTGCNGNNNNANNANNNNNNNNNNGANNTNNNCNNGGNNNNNNNNNNNNANTNNNNGNNNNNNNNNNATNNNNNNNNNNNNNCNNNTCCGGG

>21122_D01_13_14_M13R

NNNNNNNNNNNNTNAACCCTCACTAAAGGGACTAGTCCTGCAGGTTTAAACGAATTCGCCCTTCCTGTTAAGGGCAGTTTTGGTATATAAATAAAACAAGAAGCTCACTTGTCTTTATATATCTACCAAATCCAAGACATGCACCAGTGAAAGATCACAGAGAGAGAGACAAGGGGGCAGATAGATATGGATCCGGAGGCTTTCACGGCGAGTTTGTTCAAATGGGACACGAGAGCAATGGTGCCACATCCTAACCGTCTGCTTGAAATGGTGCCCCCGCCTCAGCAGCACCGGCTGCGGCGCTTGCTGTAAGGCCAAGGGAGCTATGTGGGCTAGAGGAGTTGTTTCAAGCTTATGGTATTAGGTACTACACGGCAGCGAAAATAGCTGAACTCGGGTTCACAGTGAACACCCTTTTGGACATGAAAGACGAGGAGCTTGATGAAATAATGAATAGTTTGTCTCAGATCTTTAGGTGGGATCTTCTTGTTGGTGAGAGGTATGGTATTAAAGCTGCTGTTAGAGCCGAAAGAAGAAGGCTTGATGAGGAGGATCCTAGGCGTAGGCAATTGCTCTCTGGTGATAATAATACAAATACTCTTGATGCTCTCTCCCAAGACGGTTTGGTTAGCATTGATTCTACCTTTTAGTGTAATTAAGCTAAGCTCATACTATTACTAGCTATAGGAGGCCATGGCCGTTTTATATTTCCCTTTTTGGTCGTAGTTTTATAGAGTAAATTAATTCTATGTATACTTGGATAAGATAATTAGCTTATTATNAGTTGTTACTTGCCAGCTAAGGGCGAATTCGCGGCCGCTAAATTCAATTCGCCCTATAGTGAGTCGTATTACAATTCACTGGCCGTCGTTTTACAACGTCGTGACTGGGAAAACCCTGGCGTTACCCACTTAATCNCCTTGCAGCACATCCCCCTTTCGCCAGCTGGNGNTATAGCNAAGAGGCCCGCACCGAATCGCCCTTCCCAACAGTTGCGCAGCCTATNNCGTACGGCAGTTNNNTTTANNCCTTATTAAANAAGANANTCGTTTATCGNNTGTTTGTGAATGTACAGAANNGGNNNTATGNNNACGCCGGGGNNANGATGNNANCCCCNNNCCNGTNGCACNNNTNTGCNNTGCANANNAANNNCTNACNCNNNAANNTTNNNCGGGNGNGGTGNNNNNN

>21122_E01_13_17_M13R

NNNNNNNNNNNNNNNNNTAACCCTCACTAAAGGGACTAGTCCTGCAGGTTTAAACGAATTCGCCCTTAGCTGGCAAGTAACAACTTATAATAAGCTAATTATCTTATCCAAGTATACATAGAATTAATTTACTCTATAAAACTACAACCAAAAGGGAAATATAAAACGGCCATGGCCTCCTATAACTAGTAATAGTATGAGCTTAGCTTAATTACACTAAAAGGTAGAATCAATGCTAACCAAACCTTCTTGGGAGAGAGCATCAAGAGTATTTGTATTATTATCACCAGAGAGCAATTGCCTACGCCTAGGATCCTCCTCATCAAGCCTTCTTCTTTCAGCTCTAACAGCAGCTTTAATACCATACCTCTCACCAACAAGAAGATCCCACCTAAAGATCTGAGACAAACTATTCATCATTTCATCAAGCTCCTCGTCTTTCATGTCCAAAAGGGTGTTCACTGTGAACCCGAGTTCAGCTATTTTCGCTGCCGTGTAGTACCTAATACCATAAGCTTGAAACAACTCCTCTAGCCCACATAGCTCCCTTGGCCTTACAGCAAACGCCGCAGCCGGTGCTGCTGAGGCGGGGGCACCATTTCAAGCAGACGGTTAGGATGTGGCACCATTGCTCTCGTGTCCCATTTGAACAAACTCGCCGTGAAAGCCTCCGGATCCATATCTATCTGCCCCCTTGTCTCTCTCTCTGTGATCTTTCACTGGTGCATGTCTTGGATTTGGTAGATATATAAAAGACAAGTGAGCTTCTTGTTTTATTTATATACCAAAACTGCCCTTAACAGGAAGGGCGAATTCGCGGCCGCTAAATTTCAATTCGCCCTATAGTGAGTCGTATTACAATTCACTGGCCGTCGTTTNNNAACGTCNTGACTGGGAAAACCCTGGCGTTACCCAACTTAATCGCCTTGCAGCACATCCCCCTTTCGCCAGCTGGCGTATAGCGAAGAGGCCCGCACCGATCGCCCTTTCCCAACAGTTGCGCAGCTATACGTACGNAGTTAAGGNTTANNCCTATAAAGANAGAANCCGTTATCGTCTGTTGGTGGAANGTACAGANNGATATAANTGANANNNNNNNNAANGGATNGGTGNATCCCCCTNGGCCCANNGNNACNNNNTG

>21122_F01_13_23_M13R

NNNNNNNNCNTANNATTACCCTCACTAAAGGGACTAGTCCTGCAGGTTTAAACGAATTCGCCCTTCCTGTTAAGGGCAGTTTTGGTATACAAATAAAACAAGAAGCTCACTTGTCTTTATATATCTACCAAATCCAAGACATGCACCAGTGAAAGATCACAGAGAGAGAGACAAGGGGGCAGATAGATATGGATCCGGAGGCTTTCACGGCGAGTTTGTTCAAATGGGACACGAGAGCAATGGTGCCACATCCTAACCGTCTGCTTGAAATGGTGCCCCCGCCTCAGCAGCACCGGCTGCGGCGTTTGCTGTAAGGCCAAGGGAGCTATGTGGGCTAGAGGAGTTGTTTCAAGCTTATGGTATTAGGTACTACACGGCAGCGAAAATAGCTGAACTCGGGTTCACAGTGAACACCCTTTTGGACATGAAAGACGAGGAGCTTGATGAAATGATGAATAGTTTGTCTCAGATCTTTAGGTGGGATCTTCTTGTTGGTGAGAGGTATGGTATTAAAGCTGCTGTTAGAGCTGAAAGAAGAAGGCTTGATGAGGAGGATCCTACGCGTAGGCAATTGCTCTCTGGTGATAATAATACAAATACNC

Next 8 are all L1C 190

>21122_A04_190_3_M13R

NNNNNNNNNNNNNATACCCTCACTAAAGGGACTAGTCCTGCAGGTTTAAACGAATTCGCCCTTCCTGTTAAGGGCAGTTTTGGTATACAAATAAAACAAGAAGCTCACTTGTCTTTATATATCTACCAAATCCAAGGCATGCACCAGTGAAAGATCACAGAGAGAGAGACAAGGGGGCAGATAGATATGGATCCGGAGGCTTTCACGGCGAGTTTGTTCAAATGGGACACGAGAGCAATGGTGCCACATCCTAACCGTCTGCTTGAAATGGTGCCCCCGCCTCAGCAGCACCGGCTGCGGCGTTTGCTGTAAGGCCAAGGGAGCTATGTGGGCTAGAGGAGTTGTTTCAAGCTTATGGTATTAGGTACTACACGGCAGCGAAAATAGCTGAACTCGGGTTCACAGTGAACACCCTTTTGGACATGAAAGATGAGGAGCTTGATGAAATGATAAATAGTTTGTCTCAGATCTTTAGGTGGGATCTTCTTGTTGGTGAGAGGTATGGTATTAAAGCTGCTGTTAGAGCTGAAAGAAGAAGGCTTGATGAGGAGGATCCTAGGCGTAGGCAATTGCTCTCTGGTGATAATAATACAAATACTCTTGATGCTCTCTCCCAAGAAGGTTTGGTTAGCATTGATTCTACCTTTTAGTGTAATTAAGCTAAGCTCATACTATTACTAGCTATAGGAGTCCATGGCCGTTTTGTATTTCCCTTTTGGTTGTAGTTTTATAGAGTAAATTAATTCTATGTATACTTGGATAAGATAATTAGCTTATTATAAGTTGTTACTTGCCAGCTAAGGGCGAATTCGCGGCCGCTAAATTCAATTCGCCCTATAGTGAGTCGTATTACAATTTCACTGGCCGTCGTTTTACAACGTCGTGAACTGGGGAAAACCCTTGNCGTTACCCAACTTAATCGCCTTGCAGCACATCCCCCTTTCGCCAGCTGGGCNNAATANCGAAGAGGCCCGCACCGATCGCCCTTTCCCAACAGTTGCCNCANCCTATACGTNNNNAGTTTAAGGTTTTNNNNCTANNNAAGAGGANAGCCGTTATCGTCNGNNTTNTNGGATGTANNNAANTGANNTNNANTGNNANNCNCCTGGNCCGACGNNGGGTGATACCNCCCNNNNCAGNNGNNNGNTTNNNNNNNNCNAANNAAGCNTCCCCCNNNGA

>21122_B04_190_4_M13R

NNNNNNNNNANANNANCCTCACTAAAGGGACTAGTCCTGCAGGTTTAAACGAATTCGCCCTTAGCTGGCAAGTAACAACTTATAATAAGCTAATTATCTTATCCAAGTATACATAGAATTAATTTACTCTATAAAACTACAACCAAAAGGGAAATACAAAACGGCCATGGACTCCTATAGCTAGTAATAGTATGAGCTTAGCTTAATTACACTAAAAGGTAGAATCAATGCTAACCAAACCTTCTTGGGAGAGAGCATCAAGAGTATTTGTATTATTATCACCAGAGAGCAATTGCCTACGCCTAGGATCCTCCTCATCAAGCCTTCTTCTTTCAGCTCTAACAGCAGCTTTAATACCATACCTCTCACCAACAAGAAGATCCCACCTAAAGATCTGAGACAAACTATTCATCATTTCATCAAGCTCCTCATCTTTCATGTCCAAAAGGGTGTTCACTGTGAACCCGAGTTCAGCTATTTTCGCTGCCGTGTAGTACCTAATACCATAAGCTTGAAACAACTCCTCTAGCCCACATAGCTCCCTTGGCCTTACAGCAAACGCCGCAGCCGGTGCTGCTGAGGCGGGGGCACCATTTCAAGCAGACGGTTAGGATGTGGCACCATTGCTCTCGTGTCCCATTTGAACAAACTCGCCGTGAAAGCCTCCGGATCCATATCTATCTGCCCCCTTGTCTCTCTCTGTGATCTTTCACTGGTGCATGTCTTGGATTTGGTAGATATATAAAGACAAGTGAGCTTCTTGTTTTATTTGTATACCAAAACTGCCCTTAACAGGAAGGGCGAATTCGCGGCCGCTAAATTCAATTCGCCCTATAGTGAGTCGTATTACAATTCACTGGGCCGTCGTTTTACAACGTCGTGACTGGGAAAACCCTGGCGTTACCCNACTTAATCGCCTTGCAGCACATCCCCCTTTCGCCAGCTGGCGTAATAGCGAAGAGGGCCGCACCGATCGCCCTTCCCAACAGTTGCGCAGCCTATACGTACGGCAGTTAAGGTTTACACCTATAAAAGAGAGAGCCGTTATCGTCTGTTTGGTGGATGTNNCAGAGTGATNTTANTNNNACNCCGGGGCGNNGGATGNNGANCCCCCNGCNGTGNNNNGTCTGCTNNNNAGATNAAGTCTCNNNGACTTACCGNGGNGCANNNNNGGNNNAANCTNNN

>21122_G03_190_1_M13R

NNNNNNNNNNNNNNNNNNANNCTCACTAANGGGACTAGTCCTGCAGGTTTAAACGAATTCGCCCTTAGCTGGCAAGTAACAACTTATAATAAGCTAATTATCTTATCCAAGTATACATAGAATTAATTTACTCTATAAAACTACAACCAAAAGGGAAATACAAAACGGCCATGGACTCCTATAGCTAGTAATAGTATGAGCTTAGCTTAATTACACTAAAAGGTAGAATCCATGCTAACCAAACCTTCTTGGGAGAGAGCATCAAGAGTATTTGTATTATTATCACCAGAGAGCAATTGCCTACGCCTAGGATCCTCCTCATCAAGCCTTCTTCTTTCAGCTCTAACAGCAGCTTTAATACCATACCTCTCACCAACAAGAAGATCCCACCTAAAGATCTGAGACAAACTATTCATCATTTCATCAAGCTCCTCATCTTTCATGTCCAAAAGGGTGTTCACTGTGAACCCGAGTTCAGCTATTTTCGCTGCCGTGTAGTACCTAATACCATAAGCTTGAAACAACTCCTCTAGCCCACATAGCTCCCTTGGCCTTACAGCAAACGCCGCAGCCGGTGCTGCTGAGGCGGGGGCACCATTTCAAGCAGACGGTTAGGATGTGGCACCATTGCTCTCGTGTCCCATTTGAACAAACTCGCCGTGAGAGCCTCCGGATCCATATCTATCTGCCCCCTTGTCTCTCTCTGTGATCTTTCACTGGTGCATGTCTTGGATTTGGTAGATATATAAAGACAAGTGAGCTTCTTGTTTTATTTGTATACCAAAACTGCCCTTAACAGGAAGGGCGAATTCGCGGCCGCTAAATTCAATTCGCCCTATAGTGAGTCGTATTACAATTCACTGGCCGTCGTTTTACAACGTCGTGACTGGGAAAACCCTGGCGTTACCCAACTTAATCGCCTTGCAGCACNATCCCNCCTTTCGCCAGCTGGCGTAATAGCGAAGANGCCCGCACCGATCGCCCTNCTCANAGTTGCGCANCNTANTACGTACGGCAGTTTAANNTTANCANCNANNAANAGAGNNNNTATCGTNCTGNTNNNGATGTACNNAGNGNNATTATTNGANNNNCNGGNNAANGNNNNGATTCCNNNGNNCNGNGNCNNGNCTGCTGTCAANNNNNNCCNNNNNNTNNNNNNNGGNNNNNNNNNNNNNNGNNNNNGTNNNNCNNNNNNNNNNNNNGNNCGGNNNNNN

>21122_H03_190_2_M13R

NNNNNNNNNTANNNNANNCTCACTAANNGGNNTAGTCCTGCAGGTTTAAACGAATTCGCCCTTCCTGTTAAGGGCAGTTTTGGTATACAAATAAAACAAGAAGCTCACTTGTCTTTATATATCTACCAAATCCAAGACATGCACCAGTGAAAGATCACAGAGAGAGAGACAAGGGGGCAGATAGATATGGATCCGGAGGCTTTCACGGCGAGTTTGTTCAAATGGGACACGAGAGCAATGGTGCCACATCCTAACCGTCTGCTTGAAATGGTGCCCCCGCCTCAGCAGCACCGGCTGCCGCGTTTGCTGTAAGGGCAAGGGAGCTATGTGGGCTAAAGGAATTGTTTCAAGCTTATGGTATTAGGTACTACACCGCAGCCAAAATAACTGAACTCCGGTTCACAGTGAACACCCTTTTGGACATGAAAGGAGAAGAACTTGATGAAATGATTAATAATTTGGCTCAAAACTTTAAGTGGGAACTTCTTGGTGGGGAAAAGGATTGGTATTAAAGCTGCTGTTAGAGCTGAAAAAAAAAGGCTTGATGAGGAGGATCCTAGGCGTAGGCAATTGCTCTCTGGTGATAATAATACAAATACTCTTGATGCTCTCTCCCAAGAAGGTTAGGTTAGCATTGATTCTACCTTTTAGTGTAATTAAACTAANCTCATACTATTACTAGCTATAGGAGTCCATGGCCGTTTTGTATTTCCCTTTTGGTTGTAGTTTTATGAAGTAAATTAATTCTATGTATACTTGGATAAATAATTANCTTAATTATAAGTTGTAACTTGCCAGCTAAGGCNGAATTCCCGGGCCGCTAATATCCAATTCCCC

>21144_10_190_8_M13R

NNNNNNCCNNANNNNTNACCCTCACTAAAGGGACTAGTCCTGCAGGTTTAAACGAATTCGCCCTTAGCTGGCAAGTAACAACTTATAATAAGCTAATTATCTTATCCAAGTATACATAGAATTAATTTACTCTATAAAACTACAACCAAAAGGGAAATATAAAACGGCCATGGCCTCCTATAGCTAGTAATAGTATGAGCTTAGCTTAATTACACTAAAAGGTAGAATCAATGCTAACCAAACCTTCTTGGGAGAGAGCATCAAGAGTATTTGTATTATTATCACCAGAGAGCAATTGCCTACGCCTAGGATCCTCCTCATCAAGCCTTCTTCTTTCAGCTCTAACAGCAGCTTTAATACCATACCTCTCACCAACAAGAAGATCCCACCTAAAGATCTGAGACAAACTATTCATCATTTCATCAAGCTCCTCGTCTTTCACGTCCAAAAGGGTGTTCACTGTGAACCCGAGTTCAGCTATTTTCGCTGCCGTGTAGTACCTAATACCATAAGCTTGAAACAACTCCTCTAGCCCACATAGCTCCCTTGGCCTTACAGCAAACGCCGCAGCCGGTGCTGCTGAGGCGGGGGCACCATTTCAAGCAGACGGTTAGGATGTGGCACCATTGCTCTCGTGTCCCATTTGAACAAACTCGCCGTGAAAGCCTCCGGATCCATATCTATCTGCCCCCTTGTCTCTCTCTCTGTGATCTTTCACTGGTGCATGTCTTGGATTTGGTAGATATATAAAGACAAGTGAGCTTCTTGTTTTATTTGTATACCAAAACTGCCCTTAACAGGAAGGGCGAATTCGCGGCCGCTAAATTCAATTCGCCCTATAGTGAGTCGTATTACAATTCACTGGCCGTCGTTTTACAACGTCGTGACTGGGAAAACCCTGGCGTTACCCAACTTAATCGCCTTGCAGCACATCCCCCTTTCGCCAGCTGGCGTAATAGCGAAGAGGCCCGCACCGATCGCCCTTTCCCAACAGTTGCGCAGCCTATACGTACGGCAGTTTANNNTTACACCTATAAAGAGAGANCNNNTCGTCTGTTTGTGGATGTACAGANNGATATNTGACACNNNGGNCGANNNATGGTGATCCCCCTNNNNNGNACGTCTGCTGTCANATNAGNCTCCCGNGNNTTACCNGGNGGNTGCNNNNCGNNNNGAAGCTGNNCANNNNNTNNNNNCNNNNNNNNNGNNNGNNNNNNNNTNNNNNAANNNCNNNNNNNNNNNTNANNNC

>21144_11_190_12_M13R

NNNNNNNNNNANANNANCCTCACTAAAGGGACTAGTCCTGCAGGTTTAAACGAATTCGCCCTTCCTGTTAAGGGCAGTTTTGGTATATAAATAAAACAAGAAGCTCACTTGTCTTTATATATCTACCAAATCCAAGACATGCACCAGTGAAAGATCACAGAGAGAGAGACAAGGGGGCAGATAGATATGGATCCGGAGGCTTTCACGGCGAGTTTGTTCAAATGGGACACGAGAGCAATGGTGCCACATCCTAACCGTCTGCTTGAAATGGTGCCCCCGCCTCAGCAGCACCGGCTGCGGCGTTTGCTGTAAGGCCAAGGGAGCTATGTGGGCTAGAGGAGTTGTTTCAAGCTTATGGTATTAGGTACTACACGGCAGCGAAAATAGCTGAACTCGGGTTCACAGTGAACACCCTTTTGGACATGAAAGATGAGGAGCTTGATGAAATGATGAATAGTTTGTCTCAGATCTCTAGGTGGGATCTTCTTGTTGGTGAGAGGTATGGTATTAAAGCTGCTGTTAGAGCTGAAAGAAGAAGGCTTGATGAGGAGGATCCTAGGCGTAGGCAATTGCTCTCCGGTGATAATAATACAAATACTCTTGATGCTCTCTCCCAAGAAGGTTTGGTTAGCATTGATTCTACCTTTTAGTGTAATTAAGCTAAGCTCATACTATTACTAGCTATAGGAGTCCATGGCCGTTTTGTACTTCCCTTTTGGTTGTAGTTTTATAGAGTAAATTAATTCTATGTATACTTGGATAAGATAATTAGCTTATTATAAGTTGTTACTTGCCAGCTAANGGCGAATTCGCGGCCGCTAAATTCAATTCGCCCTATAGTGAGTCGTATTACAATTCACTGGCCGTCGTTTTACAACGTCGTGACTGGGAAAACCCTGGCGTTACCCAACTTAATCGCCTTGCAGCACATCCCCCTTTCGCCAGCTGGCGTAATAGCGAAGAGGCCCGCACCGATCGCCCTTCCCAACAGTTGCGCAGCCTATACGTACGGCAGTTTAAGGTTTACACTANNNGAGANAGCCGTTATCGTCTGTTTGNGNTGTACAGANNGATATTATTGACACNNNGGNNNNNNNNGNTGATCCCCCTNNNNNGNACGTCTGCTGTCAGAATAAGNCNNCCNGTGACTTACCNNNNGGNNNNNNNTNNNNNNNAANCTNNNNNNATNNCACNANNNNNNNNGNNNNCNNNNNNNCNNNNNTNNGGGNNNNN

>21144_12_190_17_M13R

NNNNNNNNNNTANNNATTANCCTCACTAAGGGACTAGTCCTGCAGGTTTAAACGAATTCGCCCTTAGCTGGCAAGTAACAACTTATAATAAGCTAATTATCTTATCCAAGTATACATAGAATTAATTTACTCTATAAAACTACACCAAAAGGGAAATATAAAACGGCCATGGCCTCCTATAGCTAGTAATAGTATGAGCTTAGCTTAATTACACTAAAAGGTAGAATCAATGCTAACCAAACCTTCTTGGGAGAGAGCATCAAGAGTATTTGTATTATTATCACCAGAGAGCAATTGCCTACGCCTAGGATCCTCCTCATCAAGCCTTCTTCTTTCAGCTCTAACAGCAGCTTTAATACCATACCTCTCACCAACAAGAAGATCCCACCTAAAGATCTGAGACAAACTATTCATCATTTCATCAAGCTCCTCGTCTTTCATGTCCAAAAGGGTGTTCACTGTGAACCCGAGTTCAGCTATTTTCGCTGCCGTGTAGTACCTAATACCATAAGCTTGAAACAACTCCTCTAGCCCACATAGCTCCCTTGGCCTTACAGCAAACGCCGCAGCCGGTGCTGCTGAGGCGGGGGCACCATTTCAAGCAGACGGTTAGGATGTGGCACCATTGCTCTCGTGTCCCATTTGAACAAACTCGCCGTGAAAGCCTCCGGATCCATATCTATCTGCCCCCTTGTCTCTCTCTCTGTGATCTTTCACTGGTGCATGTCTTGGATTTGGTAGATATATAAAGACAAGTGAGCTTCTTGTTTTATTTATATACCAAAACTGCCCTTAACAGGAAGGGCGAATTCGCGGCCGCTAAATTCAATTCGCCCTATAGTGAGTCGTATTACAATTCACTGGCCGTCGTTTTACAACGTCGTGACTGGGAAAACCCTGGCGTTACCCAACTTAATCGCCTTGCAGCACATCCCCCTTTCGCCAGCTGGCGTAATAGCGAAGAGGCCCGCACCGATCGCCCTTTCCCAACAGTTGCGCAGCCTATACGTACGGCAGTTTAANGTTTACACCTATAAANNAGAGAGCCNTATCGTCTGTTTGTGGATGTACAGANNGATATATGACACGCNNNNGANNNNGNNATCCCCCNGNNAGNGNNCGTCNGCNNTCNNAATAANGNCTCCCGNGACTTACNGGTGNNGCANNNTNNNNNGNAAGCNNGGCCATGATGACNNCNNANTNNNNANNNNNNNNNNTCNNNNATNNGNGGNNNAAANNNNNNNN

>21145_02_190_17_b_M13R

NNNNNNNAANNNNNACCCTCACTAAGGGACTAGTCCTGCAGGTTTAAACGAATTCGCCCTTAGCTGGCAAGTAACAACTTATAATAAGCTAATTATCTTATCCAAGTATACATAGAATTAATTTACTCTATAAAACTACACCAAAAGGGAAATATAAAACGGCCATGGCCTCCTATAGCTAGTAATAGTATGAGCTTAGCTTAATTACACTAAAAGGTAGAATCAATGCTAACCAAACCTTCTTGGGAGAGAGCATCAAGAGTATTTGTATTATTATCACCAGAGAGCAATTGCCTACGCCTAGGATCCTCCTCATCAAGCCTTCTTCTTTCAGCTCTAACAGCAGCTTTAATACCATACCTCTCACCAACAAGAAGATCCCACCTAAAGATCTGAGACAAACTATTCATCATTTCATCAAGCTCCTCGTCTTTCATGTCCAAAAGGGTGTTCACTGTGAACCCGAGTTCAGCTATTTTCGCTGCCGTGTAGTACCTAATACCATAAGCTTGAAACAACTCCTCTAGCCCACATAGCTCCCTTGGCCTTACAGCAAACGCCGCAGCCGGTGCTGCTGAGGCGGGGGCACCATTTCAAGCAGACGGTTAGGATGTGGCACCATTGCTCTCGTGTCCCATTTGAACAAACTCGCCGTGAAAGCCTCCGGATCCATATCTATCTGCCCCCTTGTCTCTCTCTCTGTGATCTTTCACTGGTGCATGTCTTGGATTTGGTAGATATATAAAGACAAGTGAGCTTCTTGTTTTATTTATATACCAAAACTGCCCTTAACAGGAAGGGCGAATTCGCGGCCGCTAAATTCAATTCGCCCTATAGTGAGTCGTATTACAATTCACTGGCCGTCGTTTTACAACGTCGTGACTGGGAAAACCCTGGCGTTACCCAACTTAATCGCCTTGCAGCACATCCCCCTTTCGCCAGCTGGCGTAATAGCGAAGAGGCCCGCACCGATCGCCCTTCCCANAGTTGCGCAGCCTATACGTANNNAGTTTNNNTTACACTATAAANAAGAGANCNTNTCGGTCTGNNGNGNTGTACNGAGTGATNTNNTGANNCNNNGNNANGANGGNGATCCCCNNNNNNNNNNNNNNTGCNNTCNATAANNNNCNNNNNNTNNNNNGTGGNGNNANNNNNNANNNNNNTGGNNANGATNNTACNNNCNNNNNNNNNNNNCNNNNNGNNNAGTGCGNATCTANNNNNNN

AG alleles

>WT_AG1_717

GCTAGACTGCAGCT**ATG**GAATATCAAAATGAATCCCTTGAGAGCTCCCCCCTGAGGAAGCT**R**GGAA**GGGGAAAGGTGGAGATCAAG**CGGATCGAGAACACCACCAATC**GCCAAGTCACTTTCTGCAAA**AGGCGCAGTGGTTTGCTCAAGAAAGCCTACGA**R**TTATCTGTTCTTTGCGATGCTGAGGTTGCACTCATCGTCTTCTCTACCCGCGGTCGCCTTTATGAGTACTCTAACGATAGGTAAATAAATCTAATTTTAGATATATGCTTCTCTGGATCTTAAATTCTCCATGTTACAAGCCCTCT

>20641_C01_A1A2C316_AG_seq_F1

GNNNNNNNNNNNNNNATTNNNAATGAATCCCTTGAGAGCTCCCCCCTGAGGAAGCTAGGGAAGGGGAAAGGTGGAGATCCAAAAGGCGCAGTGGTTTGCTCAAGAAAGCCTACGAATTATCTGTTCTTTGCGATGCTGAGGTTGCACTCATCGTCTTCTCTACCCGCGGTCGCCTTTATGAGTACTCTAACGATAGGTAAATAAATCTAATTTTAGATATATGCTTCTCTGGATCTTAAATTCTCCATGTTACAAGCCCTCTTCATGTGGTCACTGTTA

>22709_02_A2C27BF_AG1_seq_F1

NNNNNNNNNNNTNNNNNGNNGCTNTGGGNATATNNNTANNNNAATCCCTTGAGAGCTCCCCCCTGAGGAAGCTGGGAAGGGGAAAGGTGGAGATCCAAGCGGATCGAGAACACCACCAATCGCCAAGTCACTTTCTGCAAAAGGCGCAGTGGTTTGCTCAAGAAAGCCTACGAGTTATCTGTTCTTTGCGATGCTGAGGTTGCACTCATCGTCTTCTCTACCCGCGGTCGCCTTTATGAGTACTCTAACGATAGGTAAATAAATCTAATTTTAGATATATGCTTCTCTGGATCTTAAATTCTCCA

>22709_03_A2C27TF_AG1_seq_F1

NNNNNNNNNNNNNNNNNNGGCANNNTNTGGNNTATCNNAATGAATCCCTTGAGAGCTCCCCCCTGAGGAAGCTGGGAAGGGGAAAGGTGGAGATCTCCGACGGGCTTCCTGGAAATCACCTCTCAAGCTTTATGCCATCACTTCCATGGATGACTTATTCTTACTTTACTGCGTCTTTGTATTTTTCTAACTTTATAGTATTACCAGTGGAAACCTCCACTGCCGCTAAATTTGATACCTTATTTTCCTGTCACCTTCCCTATCTTTATCAAGGTAGAAGAAGAAGAAGAACAAAGATTCCTCATN

>WT_AG2_717

GCTAGCAGCAGCT**ATG**GCATACCAAAATGAATCCCAAGAGAGCTCTCCCCTGAGGAAGCTGGG**R**A**GGGGAAAGGTGGAGATCAAG**CGGATCGAGAACACCAC**M**AATC**GYCAAGTCACTTTCTGCAAA**AGGCGGAATGGTTTGCTCAAGAAAGCCTATGAATTATCTGTTCTTTGCGATGCTGAGGTTGCACTCATCGTCTTCTCCAGCCGTGGACGCCTTTATGAGTACTCTAACAATAGGTATATACTTAGTTCCTC**W**GCTCATGAATTCTCCATGTTGCAA**R**CCCTCTTCAAGTGCTCACAGTTGGTTTTTCTTGCTT**Y**CTCAT**Y**CAAAGGGATTTGTTTTTT**YY**TTTT

717 AG2 from 27

>22266_19_A2C27A2F_AG2_seq_F1

NNNNNNNNNNNNTANNCNGCANCTATGGCATACCAAAATGAATCCCAAGAGAGCTCTCCCCTGAGGAAGCTGGGGAGGGGAAAGGTGGAGATAAGCGGATCGAGAACACCACCAATCGTCAAGTCACTTTCTGCAAAAGGCGGAATGGTTTGCTCAAGAAAGCCTATGAATTATCTGTTCTTTGCGATGCTGAGGTTGCACTCATCGTCTTCTCCAGCCGTGGACGCCTTTATGAGTACTCTAACAATAGGTATATACTTAGTTCCTCTGCTCATGAATTCTCCATGTTGCAAACCCTCTTCAAGTGCTCACAGTTGGTTTTTCTTGCTTCCTCATCCAAAGGGATTTGTTTTTTCCTTTTGGTTATGGCAGTGGCAATTTTTATTGGTTTTGGTTTGAACTGGTTCTTTAATTGGGTTTCTTCCCTCATCCATTTCTTTCTTCCATTGGGTTTCAACTTTTGGTGGGGGGGAAAAAAAAAAANAGGACCGGGGGGAAGGGGNNTTTA

>22186_08_A1A2C316_AG2_seq_F1

NNNGGGNNNGNTNGNNNNCAGCTATGGCATACCAAAATGAATCCCAAGAGAGCTCTCCCCTGAGGAAGCTGGGAAGGGGAAAGGTGGAAGCGGATCCAGAACCCCCCCAATCGTCCAGTCACTTTCTGCAAAAGGGGGAATGGGTTGCTCAAGAAAGCCTATGAATTATCTGGTCTTTGCGATGCTGAAGGTGCACTCCTCGTCTTCTCCAGCCGTGGACGCCTTTATGAGTACTCTAACAATAGGGATATACTTAGTTCCTCTGCTCATGAATTCTCCATGGTGCAAACCCTCTTCAAGTGCTCACAGTTGGGTTTTCTTGCTTCCTCATTCCAAGGGATTTGGTTTTTCTTTTTGGTTATGTCAGGGGCAAATTTTATTGGTTTTGTTTTTAGCTGGTTCTTTTAATGGGTTTCTTTCCTCCTCGAATTCTTTTTTTNATTGGGTTTCCACTTTTGTTGGGGGGGGAAAAAAAAAAAAAAAGGGGGGGGNAGGGNAAANAAAAAAA
